# Engineered microtissue systems for identifying the roles of Wnt and YAP signaling in hepatoblast differentiation and organization

**DOI:** 10.1016/j.mtbio.2026.103351

**Published:** 2026-06-11

**Authors:** Brock Grenci, Ariane Tsai, Katie Zobus, Gregory H. Underhill

**Affiliations:** Department of Bioengineering, University of Illinois Urbana-Champaign, 1406 West GreenStreet, Urbana, IL, 61801, USA

**Keywords:** Stem cell differentiation, *In vitro* systems, Wnt signaling, YAP, Biomechanical signaling, Tissue engineering

## Abstract

Liver development requires precise coordination of biochemical and biophysical cues to establish proper zonation and localized cell fate specification. However, there are limited models for studying the interactions between signaling pathways in defined microenvironmental contexts. Here, we employed complementary 2D microarray and 3D microwell platforms to systematically investigate how Wnt and YAP signaling pathways regulate hepatoblast differentiation and influence spatial patterning. siRNA-mediated knockdown of APC enhanced biliary marker expression and activated Notch signaling targets Hey1 and Hes1, while disrupting spatial organization patterns. YAP inhibition predominantly affected hepatocyte specification in 2D but dramatically inhibited biliary differentiation in 3D microtissues, revealing platform-dependent effects. Array culture analyses revealed that decreased cytoplasmic YAP levels, facilitated by YAP knockdown, were associated with a concomitant change in adherens junction protein expression. Collectively, the differential responses between 2D and 3D microtissue platforms are indicative of the context-dependence of intercellular interaction signals, with geometry-dependent effects influencing spatial distribution of differentiated cell types. Combinatorial pathway modulation demonstrated that Wnt and Notch signaling cooperatively regulate biliary fate, while YAP functions as a critical determinant through geometry-specific mechanisms. These findings highlight the application of engineered culture models for investigating the pathways that coordinate biochemical and biophysical signals during liver progenitor cell fate determination.

## Graphical abstract

**Figure:**
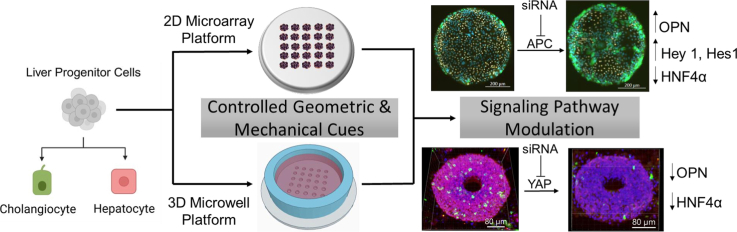

## 1 Introduction

The liver performs numerous critical functions related to metabolic homeostasis, bile secretion, and nutrient storage. To carry out such a wide range of tasks, the liver is organized into distinct functional zones with a microscale network of sinusoid and duct structures. The liver is separated into three hepatic zones: pericentral, midlobular, and periportal, each primarily comprised of hepatocytes with distinct signaling gradients responsible for determining their role [1,2]. Periportal hepatocytes are primarily responsible for bile acid synthesis, which is funneled to the intestine by the biliary tree [1,[3], [4], [5]]. Cholangiocytes, the epithelial cells that line bile ducts, also perform an extensive variety of functions and exhibit organized morphological heterogeneity [5]. Hepatocytes and cholangiocytes originate from hepatoblasts, or bipotential liver progenitor cells, and the differentiation and organization of these cells during development is crucial to ensure proper function [6]. Both liver zonation and bile duct formation are tightly controlled during embryogenesis by a complex interplay of biochemical and biophysical cues presented by the surrounding tissue microenvironment [7,8].

The Wnt/β-catenin signaling pathway is a major contributor to liver zonation and hepatic differentiation, specifically in the pericentral region where β-catenin is highly expressed. Two key regulators of this process are APC (Adenomatous Polyposis Coli), a scaffolding protein that assists in β-catenin degradation and negatively regulates Wnt signaling, and GSK3β, a kinase component of the destruction complex that phosphorylates β-catenin for degradation. Wnt ligands are secreted by liver sinusoidal endothelial cells near the central vein and inhibit the destruction complex, allowing β-catenin to accumulate and activate transcription [9,10]. While both APC and GSK3β function within the canonical Wnt pathway, APC has additional roles in cell polarity, migration, and chromosome segregation that may independently influence liver cell fate [11]. Additional pathways such as Notch, Hippo, and TGF-β signaling contribute to biliary specification and bile duct formation, highlighting the complexity of liver organization during embryogenesis [[12], [13], [14], [15]]. However, the precise role of some pathways remains unresolved; for instance, Hippo signaling, through its effector YAP (Yes-associated protein), has demonstrated context-dependent effects on liver zonation across various animal models [[16], [17], [18]]. YAP is also necessary for biliary specification and ductal morphogenesis [19,20]. While significant progress has been made in understanding biochemical regulation of liver organogenesis, much less is known about how mechanical forces influence these patterns. Disruption of the microenvironment during development or disease progression often alters mechanical signals, leading to dysregulated organization and impaired function [6]. Studying the intricate crosstalk between signaling pathways remains challenging in conventional *in vivo* and *in vitro* platforms. Engineered systems that can modulate both biophysical and biochemical cues in a well-defined manner are necessary to mimic spatiotemporal complexities and decipher the signaling interactions.

Given the limitations of *in vivo* studies, *in vitro* systems with modular microenvironmental cues serve as tools to study liver development and zonation. It has been previously reported that biophysical signals like substrate stiffness and extracellular matrix (ECM) composition can direct both hepatocytic and cholangiocytic differentiation [21,22]. For example, protein deposition onto hydrogels to create microarrays permits alteration of biophysical stimuli while concurrently modifying signaling pathways. This allows for the systematic evaluation of individual and joint effects of multiple biochemical and biophysical signals on cells. While these platforms allow for the modification of certain microenvironmental cues, 3D culture models can reproduce some *in vivo* contexts by presenting a diverse display of signals [23,24]. Stem cell-derived liver microtissue systems can model early embryogenesis and provide complex microenvironmental cues that can complement 2D platforms. Previously, we established a modular microwell system to control geometry, size, and ECM presentation to liver progenitor cell microtissues [25]. This system enables precise manipulation of physical parameters and allows for interrogation of how such cues influence cell fate. We demonstrated that the geometric cues in 3D influenced liver progenitor cell fate specification. Biophysical and biochemical modulation of liver progenitor cells in both 2D and 3D can provide insights into liver cell fate specification and assist in creating improved models for drug screening and liver disease.

In this study, we leveraged existing high-throughput 2D and 3D systems to investigate how dysregulation of Wnt and YAP signaling pathways influences liver progenitor cell fate and organization. By combining small interfering RNA (siRNA)-mediated knockdowns and small molecule inhibitors with engineered microenvironments, we systematically examined how biochemical cues interact with defined mechanical stimuli to manipulate liver cell fate. Liver progenitor cells were seeded onto 2D polyacrylamide microarrays or into 3D polyethylene glycol (PEG) acrylate microwells for analysis of cell fate and spatial patterning. Modulation of Wnt and YAP signaling exhibited geometry dependent effects on liver progenitor cell differentiation and organization, suggesting microenvironmental cues coordinately regulate cell fate and organization with these pathways.

## 2 Methods

### 2.1 Polyacrylamide hydrogel preparation

Polyacrylamide hydrogels were prepared using an adapted protocol from Tse et al. [26]. 12 mm glass coverslips (Electron Microscopy Sciences 72231-01) were etched using 0.2 M NaOH (Sigma Aldrich 415413-1L) for 1 h. The coverslips were washed 5x with dH_2_O and dried on a hot plate at 110°C. Coverslips were then silanized using 2% v/v 3-TPM in ethanol (Sigma Aldrich 440159-500 ML) for 30 min and washed 3x with ethanol before being dried on the hot plate at 110°C.

0.400g acrylamide (Sigma-Aldrich A3553-100G) and 0.040g bis-acrylamide (Sigma-Aldrich M7279-25G) were dissolved in 10 mL dH2O and filtered to synthesize 1 kPa prepolymer. Acrylamide and bis-acrylamide ratios were varied within prepolymer solutions to create 6 kPa (0.600g acrylamide and 0.045g bis-acrylamide) and 25 kPa (0.800g acrylamide and 0.055g bis-acrylamide) substrates. This prepolymer was mixed with Irgacure 2959 (BASF 55047962, 0.2 g/mL in methanol) at a 9:1 ratio. 20uL of solution was placed on a Rain-X (Rain-X 800002243) treated glass slide before placing a silanized coverslip on top of the prepolymer solution and crosslinked with UV (365 nm for 10 min). The gels were soaked in dH_2_O for 3 days to remove unreacted prepolymer and dehydrated on the hot plate at 50°C.

### 2.2 Microarray fabrication

Microarrays were created through deposition of ECM protein onto polyacrylamide hydrogels [27,28]. Polyacrylamide gels were dehydrated for at least 20 min at 50°C before ECM deposition. 384-well plates (USA Scientific Inc 18842410) containing ECM protein solutions were prepared using 2x ECM Printing Buffer (38% [v/v] glycerol (Sigma Aldrich G5516-500 ML) in dH2O, 16.4 mg/mL sodium acetate (Sigma Aldrich S2889-250G), 3.72 mg/mL EDTA (Sigma Aldrich ED-100G), 0.5% [v/v] Triton-X (Sigma Aldrich X100-1L), and ∼0.8% [v/v] glacial acetic acid (Sigma Aldrich 695092-500 ML) for a final pH of 4.8), dH_2_O, and ECM protein at a final concentration of 250ug/mL, mixed gently, and then centrifuged at 1000 RPM for 1 min. Collagen I (Sigma-Aldrich 08-115) was used to produce all array domains (‘islands’) throughout this study, unless otherwise stated. For ECM protein combinations, collagen 1 was the base and either glypican 3 (ACROBiosystems GP3-H5258), decorin (R&D Systems 143-DE-100), or lumican (ACROBiosystems LUMH5227) were combined with collagen 1 to create pairwise combinations with each protein having a final concentration of 125ug/mL. A robotic benchtop microarrayer (OmniGrid Micro, Digilab) deposited the collagen I onto the hydrogels and the microarrays were left in humidity (65% RH) overnight. Microarrays were then transferred to a 24 well plate and kept at room temperature away from light for 2 days prior to use.

### 2.3 Microwell fabrication

Microwells with spheroid and toroid geometries were fabricated following the procedure described by Berg et al. [25]. 10% w/v 4arm PEG acrylate (Laysan Bio 4arm-PEG-ACRL-10k-5g) and 0.1% w/v LAP (Sigma-Aldrich 900889-1G) were prepared in sterile PBS. A PDMS microwell mold and gasket were cleaned using IPA, and the gasket was placed around the microwell mold. 80uL of solution was placed on the PDMS mold and gently spread using a pipette tip. Bubbles were removed, and a silanized coverslip was placed on top of the solution. The mold was placed 2.5 inches under the OmniCure S1500 UV system for 30 s at 40% power (67.62 mW/cm^2^). After crosslinking, the PEG microwell was removed from the mold and placed in a 24 well plate and submerged in 1x PBS.

### 2.4 Cell culture

BMEL 9A1 cells between passages 32 and 36 were used for all experiments. BMEL cells were cultured as previously described [29,30]. BMEL cells were thawed onto collagen I (0.5 mg/mL) coated tissue culture flasks with BMEL growth media and incubated at 37°C and 5% CO_2_. BMEL growth media was made of RPMI 1640 (Corning 10-040-CV) with 10% v/v fetal bovine serum (FBS) (VWR 45000-734), 1% penicillin/streptomycin (Cytiva SV30010), 1% L-glutamine (Cytiva SH30034.01), 10ug/mL Human Recombinant Insulin (Gibco 12585014), 30 ng/mL IGF-2 (PeproTech 100-12), and 50 ng/mL EGF (PeproTech AF-100-15). Cells were either passaged or seeded onto the platforms once 70-80% confluency was reached using 0.25% v/v trypsin-EDTA (Cytiva SH30042.02) for 5 min.

siRNA treatment followed the guidelines from Horizon Discovery's Dharmacon Transfection Protocol. Cells were transfected with 25 nM siRNA using DharmaFECT 1 Transfection Reagent (T-2001-03) for 48 h in undifferentiated growth conditions prior to microarray and microwell studies. siRNA and transfection reagent were separately incubated in serum free and penicillin/streptomycin free media for 5 min. The solutions were then mixed and incubated at room temperature for 20 min before adding BMEL growth media without penicillin/streptomycin. After 48 h, cells were lifted using trypsin as described above and seeded onto the platforms. APC (J-043292-09-0005), YAP (J-046247-09-0005), CDH1 (J-041028-05-0005), and NTP (D-001810-10-05) siRNA were designed by and obtained from Horizon Discovery using their SMARTselection algorithm to generate sequences [31,32].

For microarray seeding, 1 × 10^5^ cells were placed in each well and allowed to attach for 6 h, shaking gently every 20 min for the first 2 h to ensure islands were confluent. After 6 h, the arrays were washed with 1x PBS, and differentiation media was added. Differentiation media consists of Advanced RPMI 1640 (Gibco 12633012) with 2% v/v FBS, 0.5% P/S (penicillin/streptomycin), 1% L-glutamine, and 1% MEM Non-Essential Amino Acids (Gibco 11140035). 3uM CHIR99021 in DMSO (R&D Systems 4423/10), 5uM DAPT in DMSO (Sigma-Aldrich D5942-5 MG), 250 nM verteporfin in DMSO (Sigma-Aldrich SML0534-5 MG), 2uM PRI-724 in DMSO (Selleck Chemicals S8968), and 5uM IWP-2 in DMSO (Tocris Bioscience 3533/10) were added at the start of differentiation for applicable experiments. DMSO was kept equal to or under 0.1% of the total media volume in all drug studies. Media was changed after 24 h, and cells were fixed for 15 min using 4% PFA (Electron Microscopy Sciences 15710) in PBS after 72 h of differentiation.

For microwell seeding, cells were resuspended at a concentration of 1 × 10^6^ cells/mL after lifting from the tissue culture flask. 25uL of cell solution was added to each microwell and centrifuged at 100g for 30 s then repeated once more for a total of 5 × 10^5^ cells per microwell. Growth media was added, and cells were allowed to self-aggregate overnight to form microtissues before washing and adding differentiation media. The microtissues were fixed for 30 min using 4% PFA in PBS after 72 h of differentiation.

### 2.5 RT-qPCR

Pre-differentiated cells were lifted off using trypsin, centrifuged at 800 RPM for 5 min, and lysed in 350uL of RLT Plus Lysis Buffer from the RNeasy Plus Mini Kit (Qiagen 74134). For differentiated cells on the microarrays, arrays were washed 2x with PBS and cells attached to the exposed glass on the edge of the coverslip were scraped away. Coverslips were then transferred to a new 24 well plate and lysed for 10 min on ice in RLT Plus Lysis Buffer. Cell lysate from 6 coverslips of the same condition was combined to create one biological replicate. RNeasy Plus Mini Kit was then utilized for RNA isolation. A plate reader (BioTek Synergy HTX) was used to measure RNA concentration and iScript Reverse Transcription Supermix (Bio-Rad 1708841) was used for cDNA synthesis in the Bio-Rad CFX Connect Real-Time PCR Detection System. SsoAdvanced Universal SYBR Green Supermix (Bio-Rad 1725271) and primer pairs (Supplementary Table 1) were used for cDNA amplification. Gene expression was normalized to HPRT and relevant negative controls, including either DMSO or non-targeting siRNA control (siNTP).

### 2.6 Immunocytochemistry

Protocols for immunostaining were adapted from previously described procedures [25,33,34]. Microarrays were permeabilized for 1 h using 0.25% Triton-X in PBS after fixation. Samples were then blocked for 1 h using 1% Bovine Serum Albumin (Sigma-Aldrich A2153-100G) in PBS (blocking buffer). Primary antibody solutions were prepared in blocking buffer and used to treat samples overnight at 4°C. Primary antibodies include mouse anti-HNF4α (1:200 Abcam ab41898), goat anti-OPN (1:50 R&D Systems, AF808), goat anti-E-Cadherin (1:50 R&D Systems AF748), rabbit anti-β-Catenin (1:100 Abcam ab16051), rabbit anti-YAP (1:50 Cell Signaling Technology 14074S), goat anti-Albumin (1:100 Bethyl Laboratories A90-134A), and rabbit anti-Sox9 (1:200 Millipore Sigma AB5535). Cells were treated with 10ug/mL of Brefeldin A (Tocris 1231) for 2 h prior to fixation for all experiments treated with anti-OPN or anti-Albumin. After incubation in primary antibody solution, samples were washed 3x for 5 min each and secondary antibodies in blocking buffer were added for 1 h: DyLight 550-conjugated donkey anti-mouse IgG (1:50 Abcam, ab98767), DyLight 488-conjugated donkey anti-goat IgG (1:50 Abcam, ab96935), and Alexa Fluor 647 donkey anti-rabbit IgG (1:200 Abcam ab150063). Samples were washed 3x for 5 min and mounted onto glass slides using DAPI Fluoromount-G (Southern Biotech 0100-20). For microwells, samples were permeabilized overnight using 0.5% v/v Triton-X in PBS. Samples were then blocked for 1 h using 1% Bovine Serum Albumin in PBS. Primary antibody solutions were prepared in blocking buffer and used to treat samples overnight at 4°C. Samples were washed 3x for 15 min on an orbital shaker (∼25 RPM). Secondary antibodies, including DAPI (1:5000 Invitrogen D1306), in blocking buffer were added overnight at 4°C. Samples were then washed 3x for 15 min on an orbital shaker (∼25 RPM) and mounted using ProLong Diamond Antifade solution (Molecular Probes P36961). Solution was allowed to cure overnight and mounted onto a glass slide for imaging.

### 2.7 Traction force microscopy (TFM)

TFM dishes were prepared and samples were analyzed as previously described [35]. 5 × 10^5^ cells were seeded per dish after siRNA treatment. Cells were differentiated for 72 h and then islands were imaged using the Widefield FL-Microscope at the Institute for Genomic Biology (IGB). A brightfield image was taken of all islands and the fluorescent beads right below the gel surface before dissociation of cells. Cells were dissociated using 150uL of 1% SDS +1% BSA in PBS. Beads were imaged immediately afterwards and processed following the analysis protocols.

### 2.8 Imaging and analysis

The microarrays were imaged using the AxioScan.Z1 Slide Scanner at IGB and processed as previously described [22,33,34,36]. The 10X objective was used and a lower and upper Z-height was defined for each slide. Tile scanning was used to capture the islands and stitched together using Zen Blue. Images were exported as TIFFs for each individual channel. TIFFs were processed using ImageJ and cropped in MATLAB such that each image contained one individual island. Cell Profiler was used to identify primary objects using DAPI and measure the fluorescent intensity of each channel for all cells [37]. Secondary and tertiary objects were used to identify cytoplasmic intensity and nuclear to cytoplasmic ratio based on the primary objects. A histogram was created by binning the intensity values and the point of inflection was established as the positive cutoff for staining. The point of inflection was determined by calculating a line from the bin with the highest count to the bin with the highest intensity and finding the intensity value that is furthest from the line. Isotype controls, Mouse IgG2a Isotype (Thermo Fisher 02-6200) and Goat IgG Isotype (Thermo Fisher 02-6202), were also used to establish positive cutoffs for HNF4α and OPN stains. The IgG isotype controls were used in place of the primary antibodies and the procedure in section 2.6. was followed. The mean expression and standard deviation of the control islands were used to create cutoff values. Data is exported as a CSV and processed in RStudio and GraphPad Prism 10 was used for visualization.

Microtissues were imaged using the Zeiss LSM 880 Confocal Laser Scanning Microscope at IGB. IMARIS was used for 3D reconstruction of Z-stacks and cell identification and segmentation. Data was exported and processed in R for visualization. For radial plots of microtissues, position data was used to fit a plane to each shape via PCA. For toroids, major and minor radius (R and r) were estimated and the shortest distance to the surface was calculated. For spheres, radius (R) was calculated assuming a disk-like geometry and the shortest distance to a surface was calculated.

### 2.9 Statistical analysis

Each replicate, or coverslip, in the microarray experiments is the average of 15-25 technical replicates, or islands. For the microwell system, each microtissue is a replicate. Statistical analysis was done using unpaired *t*-test, one-way analysis of variance (ANOVA), and two-way ANOVA with Tukey's multiple comparisons test in GraphPad Prism 10.

## 3 Results

### 3.1 Dysregulation of Wnt and YAP signaling modulate liver cell differentiation

**Fig. 1:**
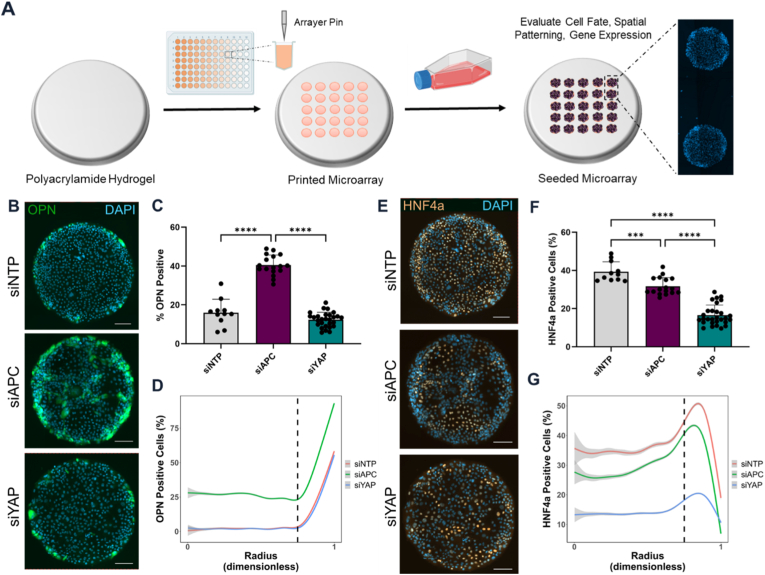
OPN and HNF4α expression are influenced by Wnt and Hippo Signaling. (A) Schematic depicting microarray fabrication and cell seeding. (B) Representative images of differentiated islands treated with siRNA targeting APC and YAP stained for DAPI (blue) and OPN (green). (C) Percent OPN+ cells for siRNA knockdowns. (D) Spatial patterning graph of OPN+ cells on the island with radius, R, being 0 at the center of the island, and R = 1 being the perimeter. The region cutoff at R = 0.75 is represented by a dashed line. (E) Representative images of differentiated islands after siRNA-mediated inhibition stained for DAPI (blue) and HNF4α (red). (F) Percent HNF4α+ cells after differentiation. (G) Spatial patterning graph of HNF4α+ cells after differentiation. P-values indicated for p < 0.05 (*), p < 0.01 (**), p < 0.001 (***), and p < 0.0001 (****). Statistics are calculated using one-way ANOVA with Tukey's post-hoc test for bar plots and error bars represent standard deviation. Scale bars are 100 μm. Schematic generated in BioRender. (For interpretation of the references to colour in this figure legend, the reader is referred to the Web version of this article.)

To examine the effects of Wnt and YAP signaling on liver cell differentiation within tightly defined cell microenvironments, we utilized a high-throughput 2D cell microarray culture system. High-throughput microarrays were created though deposition of collagen I (Col I) protein onto 1 kPa polyacrylamide gels to mimic the stiffness of healthy liver tissue (Fig. 1A) [38]. Bipotential mouse embryonic liver cells [29,39], or BMEL cells, were seeded onto Col I islands to assess liver progenitor cell differentiation. Both siRNA-mediated knockdowns and drug inhibitions were employed to dysregulate the Wnt and YAP signaling. BMEL cells were differentiated for 72 h and then either fixed for immunostaining or lysed for RT-qPCR. Immunostaining for osteopontin (OPN), a biliary marker, and hepatocyte nuclear factor 4-alpha (HNF4α), a hepatocytic transcription factor, were used to determine cell fate after differentiation. siRNA-mediated knockdowns of both APC and YAP were confirmed using RT-qPCR before and after differentiation (Fig. S1A).

Representative images of OPN expression after siRNA-mediated inhibition of APC (siAPC) and YAP (siYAP) are shown in Fig. 1B. siAPC treatment increased OPN expression and thus shifted cells towards a biliary fate while siYAP did not change OPN expression (Fig. 1C). Both conditions exhibited the same spatial trend as the non-targeting pool control, siNTP, with a higher percentage of OPN+ cells at the edge (R > 0.75) of the island than at the center (R < 0.75) of the island (Fig. 1D). The siAPC knockdown disrupted spatial patterning in the center, though, increasing the distribution of OPN+ cells in the center from 2% to 26%. The OPN+ cells also increased in the outer region from 29% in the control to 56% for the siAPC condition, nearing 90% at the perimeter of the island (R = 1) compared to 50% at the perimeter for siNTP.

Drug treatments with small molecule inhibitors, CHIR99021 (CHIR) and verteporfin (Vert), were used to further study the effects of Wnt and YAP signaling on biliary specification by suppressing additional pathway components that were not inhibited using siRNA knockdowns. CHIR99021, a GSK3β inhibitor, activates Wnt signaling similarly to siAPC by disrupting the destruction complex [40]. Verteporfin, a YAP/TEAD complex inhibitor, prevents downstream signaling from the Hippo pathway [41]. Representative images of the effects of CHIR and Vert on OPN expression are shown in Fig. S2A. CHIR increased the number of OPN+ cells by 3.25-fold, similar to siAPC, while verteporfin does not affect OPN expression (Fig. S2B). CHIR affects spatial patterning of the progenitor cells and shifts the center of the island towards a biliary fate (Fig. S2C). Overall, the data shows that APC and GSK3β inhibition increased OPN expression and shifted spatial patterning across the cellular islands, demonstrating that destruction complex inhibition and subsequent Wnt activation promotes biliary cell fate.

Representative images of HNF4α expression are shown in Fig. 1E. siYAP treated cells have a 22.8 percentage point decrease in HNF4α+ cells while siAPC treatment resulted in a 7.6 percentage point decrease compared to the control (Fig. 1F). The remaining cells that were negative for both OPN and HNF4α markers are likely undifferentiated or incompletely differentiated hepatoblasts, as many cells express intermediate levels of these markers. We have previously demonstrated that increased cell seeding density promotes more complete hepatocytic differentiation, with most cells becoming HNF4α+ at high densities similar to what is observed in 3D microtissues [25,33]. Spatial patterning followed a similar trend across all conditions and decreased uniformly across the island for the respective siRNA conditions (Fig. 1G). Drug inhibition was used to further evaluate the roles of Wnt and Hippo signaling on hepatocytic differentiation. CHIR treatment had no effect on HNF4α expression while Vert treatment downregulated HNF4α expression (Fig. S2D and S2E). APC and GSK3β inhibition showed dissimilar results, indicating different components of the destruction complex independently affect hepatocytic fate. While siYAP and Vert exposure displayed similar effects in reducing HNF4α+ cells, siYAP treatment resulted in the greatest decrease in HNF4α expression overall.

To validate cell fate specification patterns, we assessed additional hepatocytic and cholangiocytic markers. Immunostaining revealed that siAPC treatment increased SOX9 expression, particularly in island centers (Fig. S3A–D), following a spatial pattern similar to OPN. siAPC modestly reduced albumin staining, while siYAP treatment resulted in a modest increase in albumin expression within island centers (Fig S3A, E-G), despite the overall reduction in HNF4α+ cells. The increase in albumin-positive cells with siYAP, though representing a small percentage of total cells, suggests that select cells may progress toward more mature hepatocyte states under certain YAP-depleted conditions, an intriguing observation warranting future investigation. RT-qPCR analysis confirmed siAPC-mediated upregulation of *OPN* expression, while *Sox9* and *Ggt1* (gamma-glutamyl transferase 1, a cholangiocyte marker) did not show statistically significant increases, although *Ggt1* exhibited a subtle elevation in siAPC conditions (Fig. S3H). Consistent with reduced hepatocyte marker expression, siYAP treatment downregulated *Cebpa* (CCAAT/enhancer binding protein alpha), a transcription factor expressed during hepatocyte differentiation.

### 3.2 Substrate stiffness and extracellular matrix composition cooperate with biochemical cues to control liver progenitor cell fate

The microarray system was next used to study the cooperative effects of modulating stiffness and ECM with the APC and YAP knockdown cells. 1 kPa polyacrylamide gels were used as the control to mimic a healthy liver while 6 kPa and 25 kPa gels represented a transitional and diseased liver microenvironment respectively. ECM composition was also varied and included Collagen I 100% (C1), Collagen I 50% (C1 50%), Collagen I + Glypican 3 (C1/G3), Collagen I + Decorin (C1/DC), and Collagen I + Lumican (C1/LU). Glypican 3 and lumican are upregulated in liver disease and associated with poor prognosis while decorin acts as a tumor suppressor and is downregulated in disease. Both Wnt/β-catenin and Hippo/YAP signaling are known to be affected by the microenvironmental components introduced by these arrays [[42], [43], [44], [45], [46]].

**Fig. 2:**
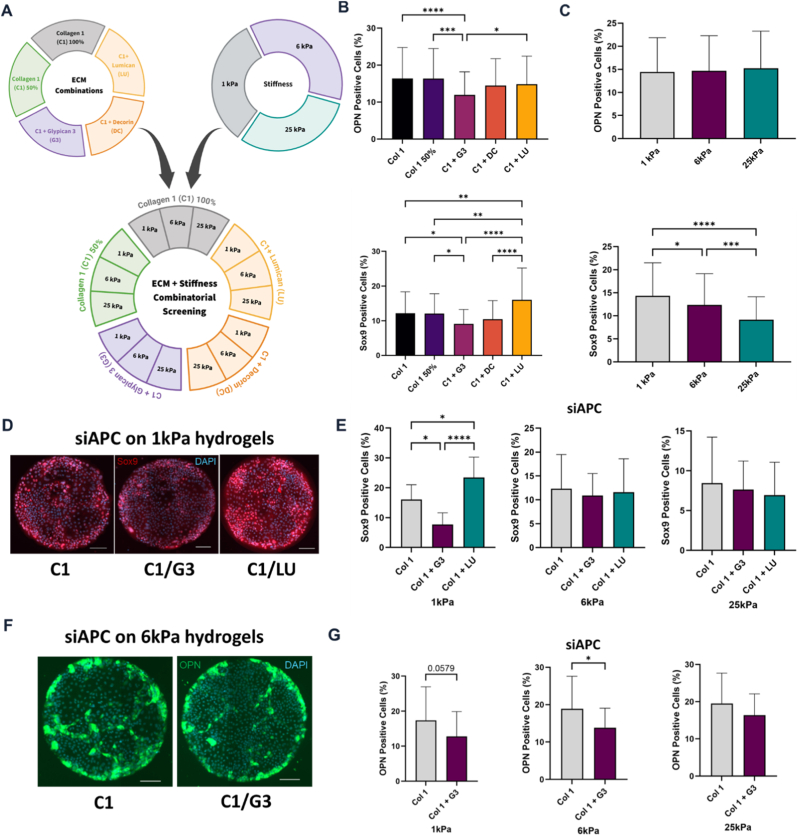
ECM and substrate stiffness modulate biliary expression (A) Schematic of microenvironmental conditions being presented using the microarray system. (B) Effect of ECM composition on percent OPN+ and Sox9+ cells for all conditions. (C) Effect of substrate stiffness on percent OPN+ and Sox9+ cells for all conditions. (D) Representative images of Sox9 expression for siAPC cells differentiated on varying ECM conditions on 1 kPa hydrogels. (E) Percent Sox9+ cells for siAPC treated cells on varying stiffnesses and ECM conditions. (F) Representative images of OPN expression for siAPC cells differentiated on varying ECM conditions on 6 kPa hydrogels. (G) Percent OPN+ cells for siAPC treated cells on varying stiffnesses and ECM conditions. Statistics are calculated using unpaired t-tests or one-way ANOVA with Tukey's post-hoc test for bar plots and error bars represent standard deviation. P-values indicated for P < 0.05 (*), P < 0.01 (**), P < 0.001 (***). and P < 0.0001 (****). Scale bars are 100 μm. Schematic made in BioRender.

Fig. 2A depicts the controlled microenvironmental cues introduced using the microarray system. Sox9 and OPN expression was evaluated to determine the effect of these cues on biliary differentiation. The introduction of glypican 3 (C1/G3) decreased OPN positive cells across all conditions from 16% to 12% when compared to collagen I islands (Fig. 2B). Similarly, C1/G3 demonstrated a decrease in Sox9 positive cells. Interestingly, the introduction of lumican (C1/LU) increases Sox9 positive cells by 1.32-fold. While both lumican and glypican 3 are upregulated in liver disease, they have distinct effects on biliary differentiation. Substrate stiffness can also alter differentiation. While stiffness had no significant effect on OPN positive cells, the percentage of Sox9 positive cells was inversely correlated with stiffness. Hydrogels with a Young's modulus of 25 kPa decreased Sox9 positive cells by 1.57-fold when compared to 1 kPa gels (Fig. 2C).

Specific combinations of stiffness, ECM composition, and signaling pathway perturbations resulted in distinct alterations. Fig. 2D shows representative images of Sox9 expression of siAPC treated cells on 1 kPa hydrogels with three different printed ECMs. On 1 kPa gels, ECM composition was able to both upregulate or downregulate Sox9 expression on C1/LU and C1/G3 islands respectively. As stiffness increases the effect of the ECM is lessened and shows no statistically significant difference when adding glypican 3 or lumican to collagen 1 on either 6 or 25 kPa gels (Fig. 2E). This demonstrates the impact a specific combination of stiffness and ECM can have on liver progenitor cell differentiation under well controlled conditions. While some combinations result in unique effects, the effect of C1/G3 islands on OPN expression was consistent across multiple conditions (Fig. 2F and G). C1/G3 reduced OPN positive cells by 1.36-fold on both 1 and 6 kPa hydrogels while 25 kPa decreased OPN positive cells by 1.19-fold when compared to collagen 1 islands on siAPC treated cells.

Hepatocytic differentiation was evaluated using HNF4α and albumin expression. Stiffness and ECM showed minimal individual effects on HNF4α positive cells while albumin positive cells increased 1.39-fold and 1.65-fold on 6 kPa gels when compared to 1 and 25 kPa substrates (Fig. S4). Overall, substrate stiffness and ECM composition have both individual and combinatorial effects on liver progenitor cell fate and can be tuned to shift differentiation of cells while simultaneously perturbing the Wnt/B-catenin and Hippo/YAP signaling pathways.

### 3.3 Wnt activation increases Notch transcription factor expression while YAP inhibition does not affect prototypical Hippo signaling target genes

**Fig. 3:**
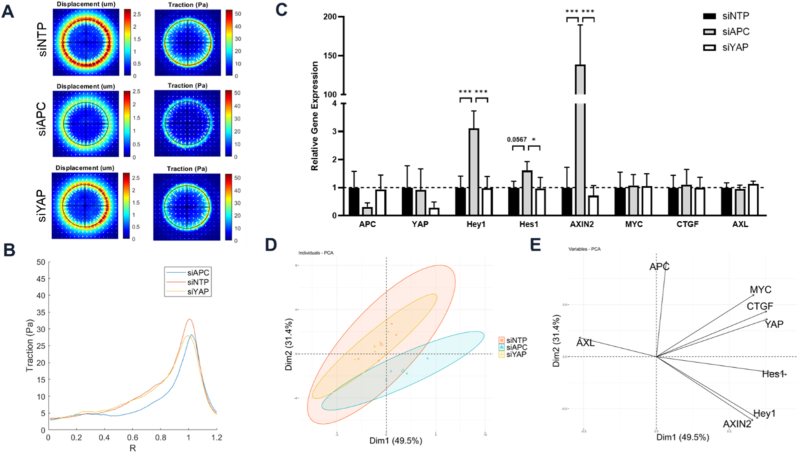
Wnt, Notch, and Hippo Pathway Gene Expression Analysis. (A) Displacement and traction force heatmaps. (B) Radial plot of traction force after differentiation. (C) Relative gene expression of siAPC and siYAP normalized to siNTP cells. (D) Biplot showing clustering of data after PCA analysis plotted on PC1 and PC2. (E) Loading vectors for the relative gene expression plotted on PC1 and PC2. P-values indicated for p < 0.05 (*), p < 0.01 (**), and p < 0.001 (***). Statistics are calculated using one-way ANOVA with Tukey's post-hoc test for bar plots and error bars represent standard deviation. Scale bars are 100 μm.

We have previously shown that the fate of these liver progenitor cells is spatially regulated by cell-cell and cell-ECM interactions, sequestering biliary cells to the edge of the islands [22]. Through our siRNA knockdown and drug treatment studies, we demonstrated that varying Wnt signaling can disrupt this spatial organization. To further investigate the underlying mechanism, we examined whether mechanical forces on the islands contribute to this disruption. Although Wnt perturbation altered cellular patterning, traction force microscopy (TFM) revealed that forces at the center of the island do not increase and cannot account for this change (Fig. 3A). Notably, siAPC- and siYAP- treated cells exhibit similar force profiles when compared to the control (Fig. 3B).

We sought to further evaluate the gene expression of the knockdown cells. Targeted genes involved in Wnt, Notch, and Hippo pathways were screened to understand the shift in spatial fate of biliary cells (Fig. 3C). siRNA-mediated knockdowns of APC caused a 138-fold increase in *AXIN2*, a downstream gene in Wnt signaling. It also showed a 3.1-fold and 1.6-fold increase in *Hey1* and *Hes1*, Notch transcription factors. A similar trend is seen with GSK3β inhibition through CHIR99021, where *AXIN2* displayed a 185-fold increase and *Hey1* and *Hes1* increase by 4.2-fold and 2.1-fold, respectively (Fig. S6A). While HNF4α expression was greatly decreased in siYAP cells at the protein level, the gene expression response showed no significant changes in Hippo target genes *AXL* and *CTGF*. YAP/TEAD complex inhibition decreased *AXL* and *CTGF* by 32% and 42%, respectively. Therefore, siYAP is regulating hepatocytic fate through another mechanism outside of Hippo signaling.

Principal component analysis (PCA) showed that the gene expression profile of siAPC cells clustered more positively on principal component 1 (PC1) and negatively on principal component 2 (PC2) when compared to the control (Fig. 3D). The loading vectors demonstrate that *AXIN2* expression is strongly associated with *Hey1* and *Hes1* expression in defining this clustering pattern (Fig. 3E), with these three genes loading together in the direction of siAPC separation from control. This demonstrates a coordinated relationship between Wnt and Notch signaling in siAPC cells, indicating that increased Wnt signaling cooperatively enhances Notch signaling to promote biliary fate.

### 3.4 Wnt and Notch signaling mediators collectively involved in biliary fate induction

**Fig. 4:**
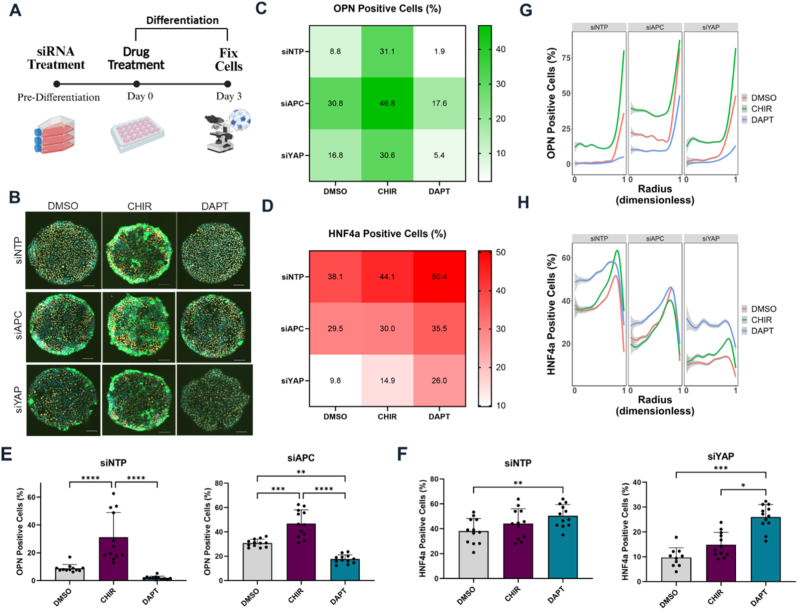
Wnt and Notch Modulation of siRNA Knockdown Cells. (A) Timeline for joint siRNA knockdown and drug treatment. (B) Representative images of islands dual treated with siRNA and drugs stained for DAPI (blue), HNF4α (red), and OPN (green) at the 72 h timepoint. (C&D) Heatmaps showing percent OPN+ and HNF4α+ cells. (E&F) Bar graphs quantifying OPN+ and HNF4α+ cells with drug treatment for certain siRNA knockdown condition. (G&H) Spatial patterning graph of OPN+ and HNF4α+ cells. P-values indicated for p < 0.05 (*), p < 0.01 (**), p < 0.001 (***), and p < 0.0001 (****). Scale bars are 100 μm. Schematic generated in BioRender. Statistics are calculated using two-way ANOVA with Tukey's multiple comparison post-hoc test for bar plots and error bars represent standard deviation. (For interpretation of the references to colour in this figure legend, the reader is referred to the Web version of this article.)

Given that we have shown Wnt modulates biliary fate and increases Hey1 and Hes1 expression, we investigated potential interactions between the signaling pathways. Combinatorial studies employing siRNA-mediated silencing and drug induced Wnt activation and Notch inhibition were used to assess Wnt, Notch, and YAP signaling crosstalk when multiple pathways are dysregulated (Fig. 4A). As Wnt signaling activates Notch transcription factors and increases OPN, DAPT was used to inhibit Notch signaling and evaluate its effect on spatial pattern restoration. As anticipated, DAPT downregulated OPN expression for all conditions and CHIR significantly upregulated OPN expression for all conditions (Fig. 4B & C). Markedly, the siAPC knockdown condition treated with DAPT displayed a reduced shift in OPN expression that more closely matched patterning of the control, showing that Notch activation is needed for siAPC knockdown to influence spatial fate of the cells (Fig. 4E & G). Conversely, treating siAPC knockdown BMELs with CHIR augmented the proportion of OPN+ cells across the island by 15% when compared to siAPC or CHIR alone. Therefore, Wnt and Notch signaling coordinately control biliary differentiation and can shift cell fate in response to the relative levels of Wnt and Notch activity. The siYAP knockdown condition displayed no changes in biliary patterning versus the control with either drug treatment. The lack of change in OPN levels and gene expression indicates that the siYAP knockdown does not play a coordinated role with either Wnt or Notch signaling on biliary differentiation in 2D. Overall, Wnt signaling activation shifts cells towards a biliary fate and simultaneous inhibition of separate parts of the destruction complex can further increase OPN expression in these cells.

The same combinatorial treatments were used to study hepatocytic fate. siYAP exhibited the greatest effect, inhibiting hepatic differentiation in all conditions by 24-29 percentage points compared to the controls (Fig. 4D). While this was the main contributing factor to the differences in hepatic fate, Wnt and Notch still played smaller roles in modulating differentiation (Fig. 4F & H). Specifically, DAPT increased HNF4α expression and was most notable in conjunction with the siYAP knockdown. While it did not rescue the HNF4α expression (p = 0.0059), it does indicate that inhibiting Notch can assist in directing cells towards a hepatic fate. The Wnt signaling modifications had contradictory effects on HNF4α expression, similar to what was seen in Fig. 1. GSK3β inhibition did not decrease HNF4α expression while siAPC did, signifying a potential role for APC outside of Wnt signaling that affects hepatic fate. Fig. S5 demonstrates that spatial organization of these liver progenitor cells occurs after array seeding and the hepatocytic and biliary fate across the island is modified by the knockdowns and small molecule inhibitors. Overall, the results indicate APC plays a role in both hepatic and biliary specification while YAP is crucial for hepatic specification in 2D.

While the roles of different components in the destruction complex provide insight into Wnt/β-catenin signaling activation, inhibition of Wnt signaling can additionally affect liver development. Specifically, while downregulation of Wnt signaling contributes to hepatic specification, maturation, and zonation, β-catenin deletion can result in decreased bile duct formation while also inhibiting hepatic markers such as HNF4α and CEBPA [47,48]. To better understand the mechanisms behind this PRI-724, an inhibitor of CBP/β-catenin binding, and IWP-2, a PORCN inhibitor, were used to disrupt Wnt signaling [49]. 2uM PRI-724 treatment suppresses expression of HNF4a, OPN, and Sox9, demonstrating a reduction in both hepatic and biliary specification across the islands. This suggests that β-catenin-mediated signaling is needed to promote differentiation. 5uM IWP-2 treatment showed no effect on OPN or HNF4α, demonstrating this minimal level of signaling may not rely on Wnt ligand secretion (Fig. S7).

### 3.5 YAP inhibition, but not YAP/TEAD complex inhibition, increases adherens junction protein

**Fig. 5:**
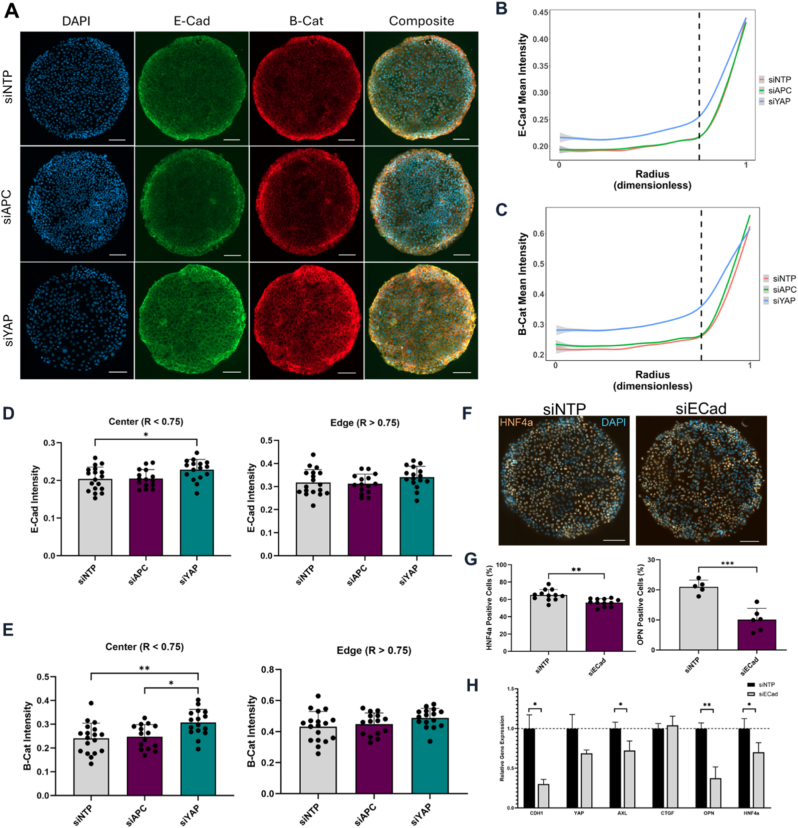
Adherens Junction Protein Expression. (A) Representative images of islands stained for DAPI (blue), β-catenin (red), and E-Cadherin (green) at the 72 h timepoint. (B&C) Radial plots showing E-Cadherin & β-catenin expression levels. (D&E) Bar graphs for E-Cad & β-Cat expression in the center region of the island versus in the edge region. P-values indicated for p < 0.05 (*), p < 0.01 (**), and p < 0.001 (***). Statistics are calculated using one-way ANOVA with Tukey's post-hoc test for bar plots and error bars represent standard deviation. Scale bars are 100 μm. (For interpretation of the references to colour in this figure legend, the reader is referred to the Web version of this article.)

In addition to the gene expression and signaling pathway relationship studies, the microarrays were applied to identify the role of cell-cell interactions in these processes. Adherens junction expression of siRNA-mediated knockdown cells was quantified using E-Cadherin and β-catenin [50,51]. The proteins are primarily localized to cell-cell junctions in the center of the island and more diffuse near the edges in all conditions (Fig. 5A). A negligible difference in the presence of both adherens junction proteins is shown in the siAPC knockdown while the siYAP knockdown demonstrates a substantial increase in both, specifically in the center of the island (Fig. 5B–E). This finding was counterintuitive, as siYAP reported a lower cell density and less hepatocytic differentiation on the islands, and we have previously show that HNF4α expression is dependent on E-Cadherin [33]. Furthermore, YAP/TEAD complex inhibition did not show the same effects on E-Cadherin or β-catenin expression levels (Fig. S8). E-Cadherin expression was unaffected while β-catenin expression was decreased. YAP plays an integral role in E-Cadherin mediated contact inhibition and has also been shown to interact with adherens junctions when phosphorylated [[52], [53], [54]]. This, in conjunction with the RT-qPCR data, indicates that YAP has a role outside of the Hippo pathway that affects hepatic fate. We hypothesize it is interacting with or affecting adherens junctions between cells.

**Fig. 6:**
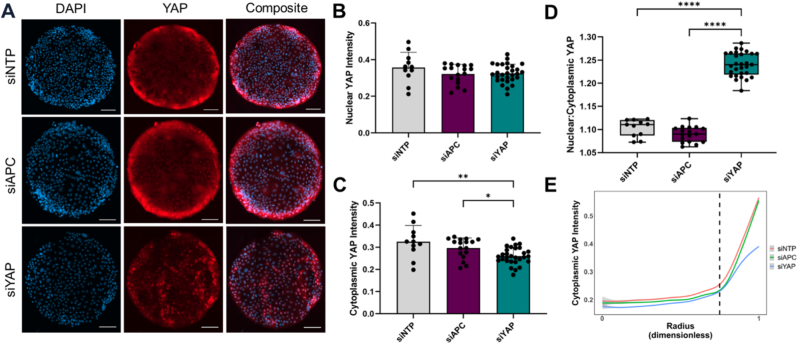
YAP Expression. (A) Representative images of DAPI (blue) and YAP (red) after siRNA treatment and differentiation. (B&C) Bar graph depicting nuclear and cytoplasmic YAP intensity for each condition. (D) Box and whisker plot for the ratio of nuclear to cytoplasmic YAP expression after differentiation. (E) Radial plot for cytoplasmic YAP expression. P-values indicated for p < 0.05 (*), p < 0.01 (**), p < 0.001 (***), and p < 0.0001 (****). Scale bars are 100 μm. (For interpretation of the references to colour in this figure legend, the reader is referred to the Web version of this article.)

siRNA mediated inhibition of E-Cadherin (siECad) was used to study the relationship between adherens junctions and Hippo/YAP signaling for hepatoblast differentiation. It has previously been shown that E-Cadherin inhibition reduces HNF4α expression [33]. Fig. 5F and G demonstrate that E-Cadherin inhibition reduces OPN and HNF4α positive cells while also reducing gene expression of *AXL* and *YAP*. This indicates that E-Cadherin inhibition affects Hippo/YAP expression and alters differentiation patterning. To further explore this, we performed immunostaining for YAP expression and quantified nuclear versus cytoplasmic levels of YAP in the siRNA treated cells (Fig. 6A).

Immunostaining revealed that nuclear expression of YAP does not change at a protein level (Fig. 6B) even though mRNA expression was shown to decrease in Fig. 3C. Cytoplasmic YAP expression did change, though (Fig. 6C). siYAP had less YAP expression overall and a significantly higher nuclear to cytoplasmic ratio of YAP expressed (Fig. 6D). This is indicative of the knockdown producing an effect; however, it did not affect nuclear YAP. The radial profile in Fig. 6E shows that cytoplasmic YAP intensity for the siYAP condition is lower than the control at both the edge and center of the island. YAP expression was not evaluated for the drug treatment conditions due to the lack of increased adherens junction protein expression in the verteporfin condition. This suggests that the siRNA knockdown decreased cytoplasmic YAP without lowering nuclear YAP, illustrating cytoplasmic YAP plays a role in hepatic specification.

### 3.6 Microtissue geometry and Wnt/YAP pathway perturbation influence resultant 3D differentiation extent and spatial patterning

**Fig. 7:**
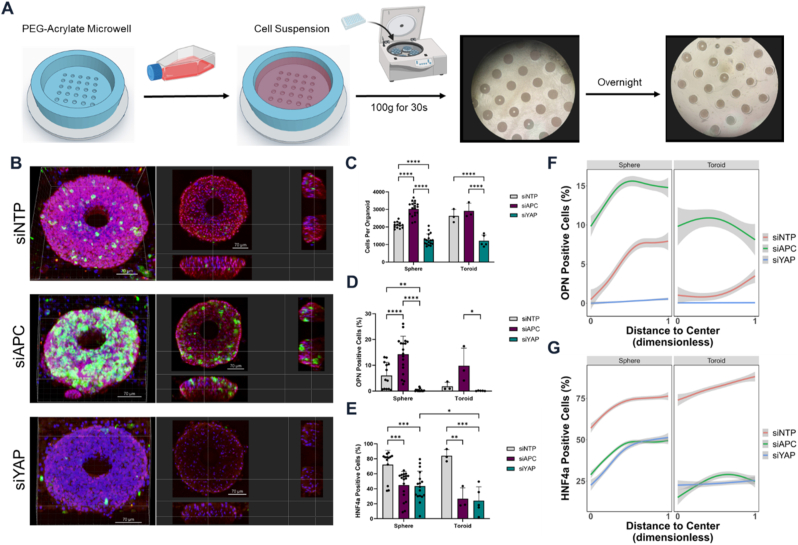
3D Microtissue Differentiation and Patterning in Microwells. (A) Schematic of microwell seeding and microtissue formation. A microwell containing both spheroids and toroids is shown in the brightfield image. (B) Representative images of toroid microtissue stained for DAPI (blue), HNF4α (red), and OPN (green). (C) Bar graph depicting cells per microtissue for each microwell shape and siRNA knockdown. (D&E) Bar graphs exhibiting the percentage of OPN+ and HNF4α+ cells in the microtissues based on geometry and siRNA knockdown. Statistics are calculated using two-way ANOVA with Tukey's post-hoc multiple comparisons test for bar plots and error bars represent standard deviation. (F&G) Radial plots of normalized distance to the surface of the microtissue (0 is surface, 1 is furthest away from all surfaces). P-values indicated for p < 0.05 (*), p < 0.01 (**), p < 0.001 (***), and p < 0.0001 (****). Scale bars are 100 μm. Schematic generated in BioRender. (For interpretation of the references to colour in this figure legend, the reader is referred to the Web version of this article.)

Next, scaffold-free 3D cellular aggregates (microtissues) were utilized to study the effect of signaling pathway modulation, using siRNA-mediated knockdown, in the context of defined multicellular geometries. 4-arm PEG acrylate microwells were fabricated and seeded with siRNA treated cells, followed by overnight incubation for self-aggregation (Fig. 7A). Differentiation began the following day, and microtissues were fixed after 72 h. Fig. 7B and C depicts a toroid-shaped microtissue that was stained for HNF4α (red), OPN (green), and DAPI (blue). Liver progenitor cell differentiation in response to 3D microtissue culture was analyzed using both spherical and toroidal geometries. siYAP treated microtissues resulted in lower cell density for each condition, with only 53% of the cell per microtissue compared to the siNTP condition across both shapes (Fig. 7C). Like the microarrays, siAPC knockdown increased OPN+ cells and reduced HNF4α+ cells in both geometries (Fig. 7D & E). siYAP microtissues did have fewer HNF4α+ cells in 3D, similar to the microarrays. Conversely, OPN expression was almost completely inhibited in 3D, whereas OPN expression on the microarrays had remained unchanged by the siYAP treatment. The geometry of the microtissue also contributed to cell fate specification. Toroids have a decrease in HNF4α+ and OPN+ cells for siYAP and siAPC cells when compared to spheroids. Alternatively, toroids have increased HNF4α+ cell for the siNTP condition.

For the siNTP control condition, HNF4α+ cells are distributed throughout the microtissue, and spatial patterning was evident with a higher concentration of HNF4α positive cells localized to the outer layer (distance = 1) (Fig. 7F). The outer layer of both the spheroid and toroid microtissues are primarily under tension based on 3D FEM models [25]. For spatial analysis, the normalized distance to surface represents the shortest distance from each cell to the outer surface of the microtissue, where distance = 0 represents the furthest point from any external surface and distance = 1 is at the surface (see Supplementary Fig. S9C for schematic). In toroidal geometries, this metric captures cells throughout the entire tissue volume, including those in the inner region facing the hollow center. A similar pattern is present for the OPN+ cells, although the toroid geometry prompted a sharper decrease in OPN expression near the microtissue's center (distance = 0) (Fig. 7G). siAPC knockdowns display the same pattern as siNTP for the spheres, but spatial organization of the cells within the toroid geometries was disrupted. OPN+ cells peak in the middle region of the siAPC toroids which is associated with higher compression based on 3D FEM models [25]. This suggests APC influences spatial organization in both 2D and 3D liver microtissue. Organization of HNF4α+ cells is also altered based on microtissue geometry for the siYAP knockdown. There is no observed spatial organization in the toroids while the spheroids follow the same trend as siNTP with higher HNF4α at the surface. These results demonstrate the cooperative role that multicellular aggregate geometry plays in coordinating the intercellular signaling regulation of cell differentiation patterning.

## 4 Discussion

We utilized high-throughput 2D and 3D microtissue systems to systematically evaluate the interplay between Wnt, YAP, and Notch signaling pathways in liver progenitor cell differentiation. Our findings reveal that inhibition of Wnt destruction complex components disrupts typical spatial organization while enhancing biliary specification through cooperative interactions with Notch signaling. YAP signaling modulation exhibits effects on hepatocyte and biliary marker expression that are dependent on culture context. These results demonstrate the necessity of integrating multiple signaling pathways with mechanical cues to understand liver cell fate specification and organization.

The enhanced OPN expression observed following APC or GSK3β inhibition suggests activation of Wnt signaling promotes biliary differentiation, consistent with previous observations that Wnt/β-catenin signaling influences bile duct development [[55], [56], [57]]. The accompanying increase in AXIN2 expression indicates negative feedback regulation of Wnt signaling, as AXIN2 serves as both a target and inhibitor of the pathway [58,59]. This regulatory mechanism may prevent excessive Wnt activation while maintaining appropriate signaling levels for biliary specification. Particularly striking was the cooperative relationship between Wnt and Notch signaling pathways in biliary specification. APC or GSK3β inhibition significantly enhanced expression of Notch downstream targets Hey1 and Hes1, indicating that Wnt activation potentiates Notch signaling activity. This crosstalk is consistent with observations in intestinal development, where APC inhibition similarly upregulates Notch signaling [60]. The ability of Notch inhibition to counteract the effects of APC inhibition further supports this cooperative model. Such pathway interactions have crucial implications for understanding bile duct formation, as both Wnt and Notch signaling are essential for proper biliary differentiation during liver development.

The differential effects of APC versus GSK3β inhibition on HNF4α expression reveal pathway-specific regulation of hepatocyte specification. While APC inhibition substantially reduced HNF4α expression, GSK3β inhibition had minimal effect, suggesting APC functions in hepatocyte differentiation through mechanisms independent of the Wnt destruction complex. The reduction in HNF4α and albumin expression is consistent with previous work demonstrating that APC affects liver zonation and hepatic fate specification [9,61]. The preservation of HNF4α expression following GSK3β inhibition indicates that canonical Wnt signaling activation alone is insufficient to suppress hepatocyte differentiation, pointing to additional APC-mediated regulatory mechanisms.

Controlled multicellular geometry, in both 2D and 3D systems, produced distinct spatial patterning effects that were further modulated by biochemical signaling. Consistent with our previous observations, defined geometric boundaries established spatial distributions of cell fates in 2D, with biliary specification associating with regions of higher traction stress at island edges [25,30], and with distinct compressive and tensile force distributions in 3D microtissue geometries predicted by finite element modeling [25]. However, Wnt pathway modulation altered these patterns, particularly in 3D microtissues where siAPC treatment shifted peak OPN expression from the surface to intermediary regions experiencing maximum compression in toroids. This spatial redistribution is consistent with geometry-imposed compressive gradients cooperating with Wnt signaling perturbation to influence biliary specification, further highlighting the importance of multicellular geometric context in evaluating signaling pathway effects. Additional cooperative microenvironmental regulation effects were demonstrated following incorporation of controlled substrate stiffness and ECM composition conditions into the microarray cultures. Glypican 3 downregulated both OPN and Sox9. Conversely, the presence of lumican upregulated Sox9 expression in combination with the knockdown of APC. Overall, these findings illustrate how the liver progenitor cells utilize Wnt/β-catenin and YAP signaling pathways to regulate differentiation patterning in the context of co-regulating ECM composition and stiffness cues. In the liver, progenitor cells contribute to the onset and progression of liver diseases such as fibrosis or liver cancer in which stiffness and ECM are also dysregulated [62], necessitating the study of the combinatorial effects of stiffness, ECM, and Wnt or Hippo signaling, using engineered culture platforms such as the microarray platform.

YAP emerged as a critical regulator of hepatocyte specification, with YAP inhibition producing the most pronounced effects on hepatic differentiation in 2D systems. The lack of effect on canonical Hippo target genes AXL and CTGF, despite reduced hepatocyte marker expression, suggests that YAP may function through non-canonical mechanisms in liver progenitors. The decreased cytoplasmic YAP levels and increased adherens junction protein expression following siYAP treatment point to a role for YAP in mechanosensing at cell-cell contacts. The loss of spatial organization in siYAP-treated toroids further supports this interpretation, as YAP-mediated responses to geometry-imposed mechanical gradients may be essential for establishing proper cell fate patterns within defined multicellular architectures. The geometry-dependent function of YAP is particularly relevant given our previous findings that cell-cell interactions promote hepatocyte specification and that E-cadherin inhibition reduces hepatic differentiation [25,33]. While inhibition of E-cadherin was confirmed to reduce HNF4α expression and decrease expression of Hippo-related genes, direct validation of junctional force-mediated signaling will require additional approaches. Techniques such as co-immunoprecipitation or FRET-based molecular tension sensors, which have been used to quantify actomyosin-generated tension transmitted through E-cadherin at cell-cell contacts [63], would enable additional assessment of the role of YAP in adherens complexes and junctional tension. Three-dimensional traction force microscopy approaches applied to encapsulated multicellular aggregates represent an additional avenue for directly quantifying cell-generated forces in 3D contexts [64]. The observation that siYAP treatment affected biliary specification differently in 2D versus 3D systems underscores the distinct mechanical environments these platforms provide. In 2D microarray cultures, where geometric confinement of islands primarily drives spatial patterning through cell-ECM interactions at pattern boundaries, YAP inhibition had minimal effect on OPN expression. In contrast, scaffold-free 3D microtissues, where geometry-imposed gradients arise predominantly through cell-cell interactions, revealed substantial effects of YAP inhibition on biliary specification. This differential response indicates that YAP function in biliary fate regulation is particularly sensitive to the geometry-dependent cell-cell interaction context provided by 3D multicellular aggregates.

The geometry-dependent effects observed in 3D scaffold-free microtissues are interpreted in the context of mechanical cue gradients that differ between spheroidal and toroidal geometries. While the current study does not directly quantify intracellular forces in 3D, prior finite element modeling studies of these microwell geometries have established that toroidal and spheroidal configurations generate distinct compressive and tensile force distributions, with toroidal interiors experiencing elevated compression relative to the surface [25]. The spatial redistribution of OPN+ cells toward intermediate regions in siAPC toroids, and the loss of HNF4α patterning in siYAP toroids, are therefore consistent with geometry-mediated mechanical gradients cooperating with these signaling perturbations to regulate cell fate. The central focus of the present work is to define how Wnt and YAP pathways regulate differentiation trajectories within controlled multicellular geometries, and the observed geometry-dependent outcomes motivate future studies that directly quantify mechanotransduction in response to pathway perturbations. Additionally, while the scaffold-free microwell platform used here enables systematic examination of cell-cell interactions within defined geometries, fully embedded 3D culture systems present additional extracellular matrix interactions that can influence cell spreading, migration, and differentiation in distinct ways [65]. The type of encapsulating biomaterial, together with its stiffness and ECM composition, represent important variables for future investigation, and PEG-maleimide-based encapsulation within these microwell geometries has been shown to influence hepatoblast differentiation, providing a potential path toward incorporating these additional cues [66].

While the engineered microtissue systems employed in this study enabled systematic investigation of Wnt and YAP signaling within controlled biophysical contexts, several additional microenvironmental factors warrant consideration in future work. The absence of liver non-parenchymal cells (NPCs), particularly liver portal fibroblasts and liver sinusoidal endothelial cells (LSECs), represents a limitation of the current model, as these NPCs are known to secrete paracrine ligands that guide cell differentiation during liver development [10,67]. LSECs improve differentiation of hepatoblasts and regulate liver regeneration through angiocrine signals such as Wnt2 [68,69]. Portal fibroblasts express Notch ligands and contribute to biliary development but have been shown to improve function of hepatocytes [[70], [71], [72]]. Additionally, gradients in oxygen tension and nutrient availability may contribute to spatial patterning within 3D microtissues, potentially working cooperatively with the mechanical compression and cell-cell signaling investigated here. Similarly, while our platforms controlled ECM presentation through collagen I deposition in 2D arrays and defined geometries in 3D PEG microwells, the complex ECM composition found *in vivo* includes various laminin isoforms, fibronectin, and other matrix proteins that influence both hepatocyte and cholangiocyte differentiation [21,22]. A key advantage of the modular microtissue platforms presented here is the ability to systematically introduce and evaluate these additional factors in isolation or combination. The controlled geometries, cell numbers, and culture timelines provide reproducible baseline conditions that are difficult to achieve in conventional culture systems, enabling future studies to directly examine how NPCs, oxygen gradients, nutrient availability, and complex ECM compositions cooperatively regulate liver progenitor cell fate specification and spatial organization alongside the Wnt, YAP, and Notch pathways characterized in this work. Encapsulation of cells in 3D can more closely mimic *in* *vivo* conditions and add additional complexity to the culture.

The complementary use of 2D and 3D platforms proved essential for dissecting the complex interactions between signaling pathways and biophysical cues. Each system provides distinct geometric contexts that reveal different aspects of pathway function and cell fate regulation. The 2D microarray platform enabled precise control of cell-ECM interactions and substrate properties, while 3D microtissues with defined spherical and toroidal geometries provided physiologically relevant cell-cell interactions and distinct mechanical contexts. The controlled geometries in 3D microtissues were particularly important for revealing how compression and spatial organization influence pathway-mediated differentiation, with toroidal geometries disrupting typical patterning in response to APC and YAP perturbation. Notably, the identification of cooperative interactions between Wnt and Notch signaling in directing biliary specification represents a key advance that provides new targets for manipulating cholangiocyte fate in engineered liver tissues. The geometry-dependent functions of YAP, particularly the differential effects observed between 2D and 3D culture contexts and the association with altered cytoplasmic YAP levels and adherens junction expression, further highlight context-specific roles for YAP in hepatocyte specification that appear to operate independently of canonical Hippo signaling. These mechanistic insights into how liver progenitors integrate biochemical pathway signals with geometric and biophysical cues establish a foundation for future investigations.

Building upon the signaling pathway interactions and geometry-dependent effects characterized here using the murine-derived bipotential liver cells, future studies can extend these findings to human pluripotent stem cell-derived hepatoblasts or primary human fetal liver progenitors. The Wnt/β-catenin, Hippo/YAP, and Notch signaling pathways are highly conserved across species, and the engineered microarray and microwell platforms developed here are directly compatible with human cell types. Such translational studies would have important implications for understanding developmental disorders affecting liver zonation and bile duct formation, including Alagille syndrome and biliary atresia, where Notch and other developmental pathways are disrupted [12,14]. The context-dependent functions of YAP identified in this work are particularly relevant to liver cancer, where aberrant YAP activation contributes to hepatocellular carcinoma and cholangiocarcinoma progression [17,18]. Additionally, the cooperative regulation of biliary fate by Wnt and Notch signaling may inform regenerative medicine strategies for generating functional cholangiocytes for bile duct repair.

Understanding how these core developmental pathways interact with defined geometric contexts may also inform therapeutic strategies for liver diseases characterized by altered tissue architecture and aberrant cell fate specification, such as fibrosis and cholangiopathies. Subsequent studies should continue to leverage both 2D and 3D platforms to comprehensively investigate how liver progenitors integrate multiple signaling inputs with geometric and biophysical cues during development, regeneration, and disease.

## CRediT authorship contribution statement

**Brock Grenci:** Conceptualization, Data curation, Formal analysis, Investigation, Methodology, Software, Validation, Visualization, Writing – original draft, Writing – review & editing. **Ariane Tsai:** Data curation, Investigation, Validation, Writing – review & editing. **Katie Zobus:** Investigation, Validation, Visualization, Writing – review & editing. **Gregory H. Underhill:** Conceptualization, Funding acquisition, Methodology, Project administration, Resources, Supervision, Writing – original draft, Writing – review & editing.

## Declaration of competing interest

The authors declare that they have no known competing financial interests or personal relationships that could have appeared to influence the work reported in this paper.

## Data Availability

Data will be made available on request.
