# Supplementary material for: Engineered microtissue systems for identifying the roles of Wnt and YAP signaling in hepatoblast differentiation and organization
Source: Mater Today Bio. 2026 Jun 11;39:103351. doi: 10.1016/j.mtbio.2026.103351 (PMC13285378; doi:10.1016/j.mtbio.2026.103351)

**Supplemental Figures and Tables**


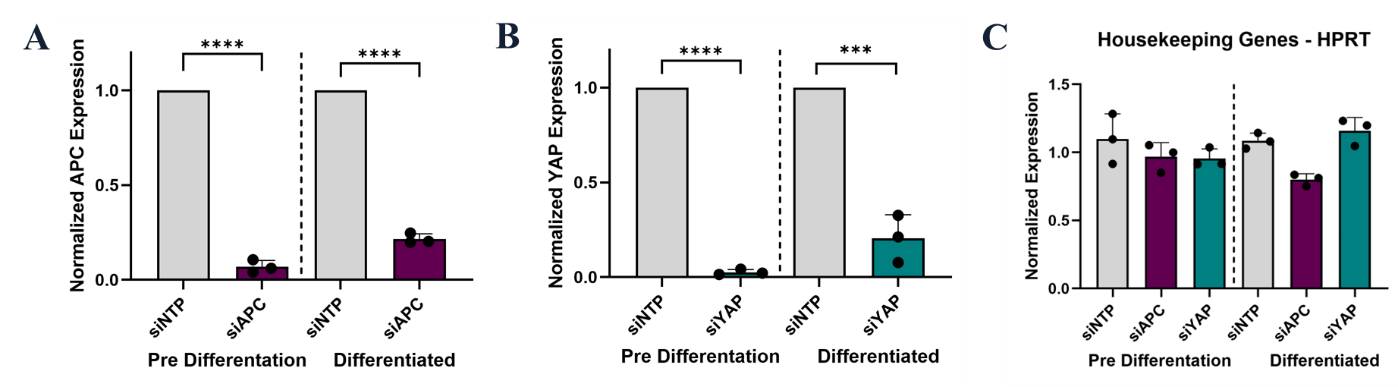


**Supplemental Figure S1** siRNA Knockdown Confirmation (A) Expression of APC after siAPC treatment pre and post differentiation (B) Expression of YAP after siYAP treatment pre and post differentiation (C) HPRT expression variability normalized for all knockdowns. P-values indicated for P < 0.05 (*), P < 0.01 (**), P < 0.001 (***), and P < 0.0001 (****).


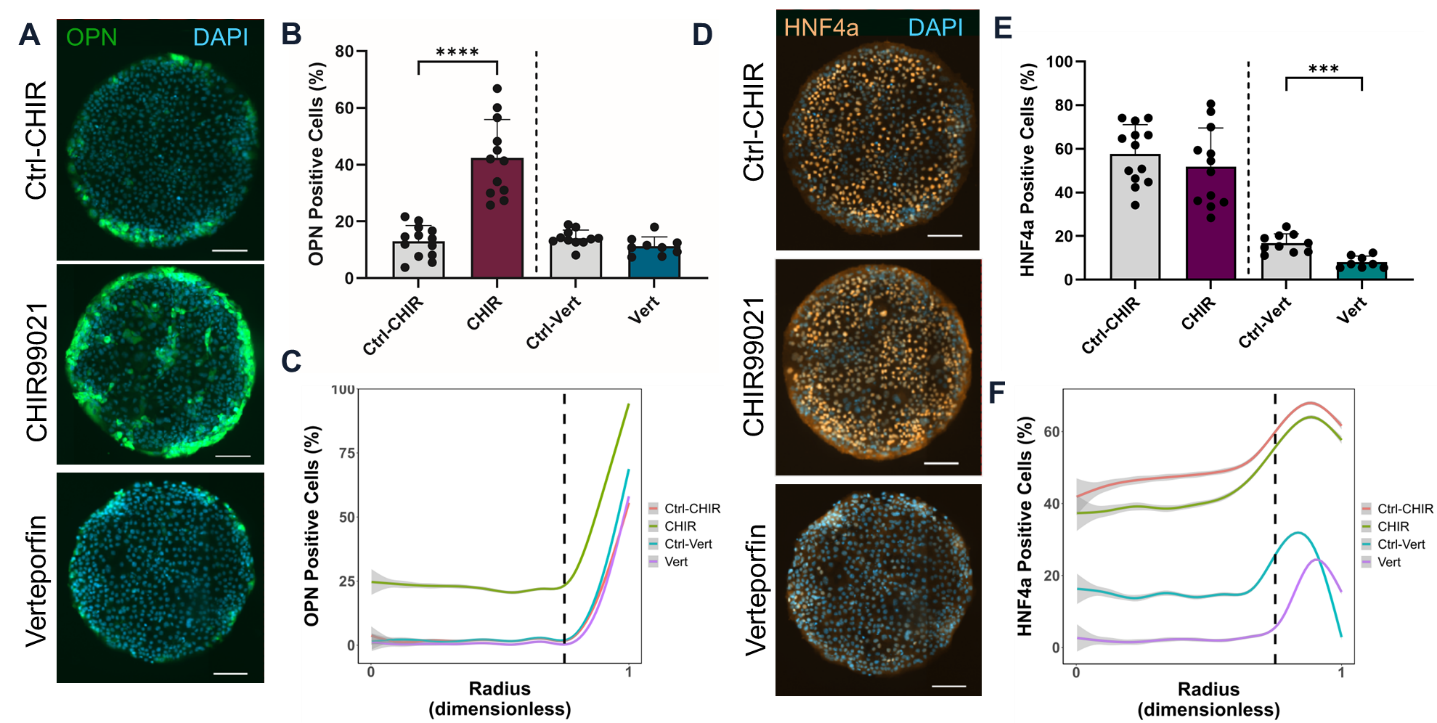


**Supplemental Figure S2** CHIR99021 and Verteporfin Treatment (A&D) Representative images of differentiated islands treated with CHIR99021 and verteporfin for 72 hours stained for DAPI (blue), OPN (green), and HNF4α (red). (B&E) Percent OPN+ and HNF4α+ cells for each drug treatment condition. (C&F) Spatial patterning graphs. Statistics are calculated using unpaired t-test for bar plots and error bars represent standard deviation. P-values indicated for P < 0.05 (*), P < 0.01 (**), P < 0.001 (***), and P < 0.0001 (****). Scale bars are 100 μm.


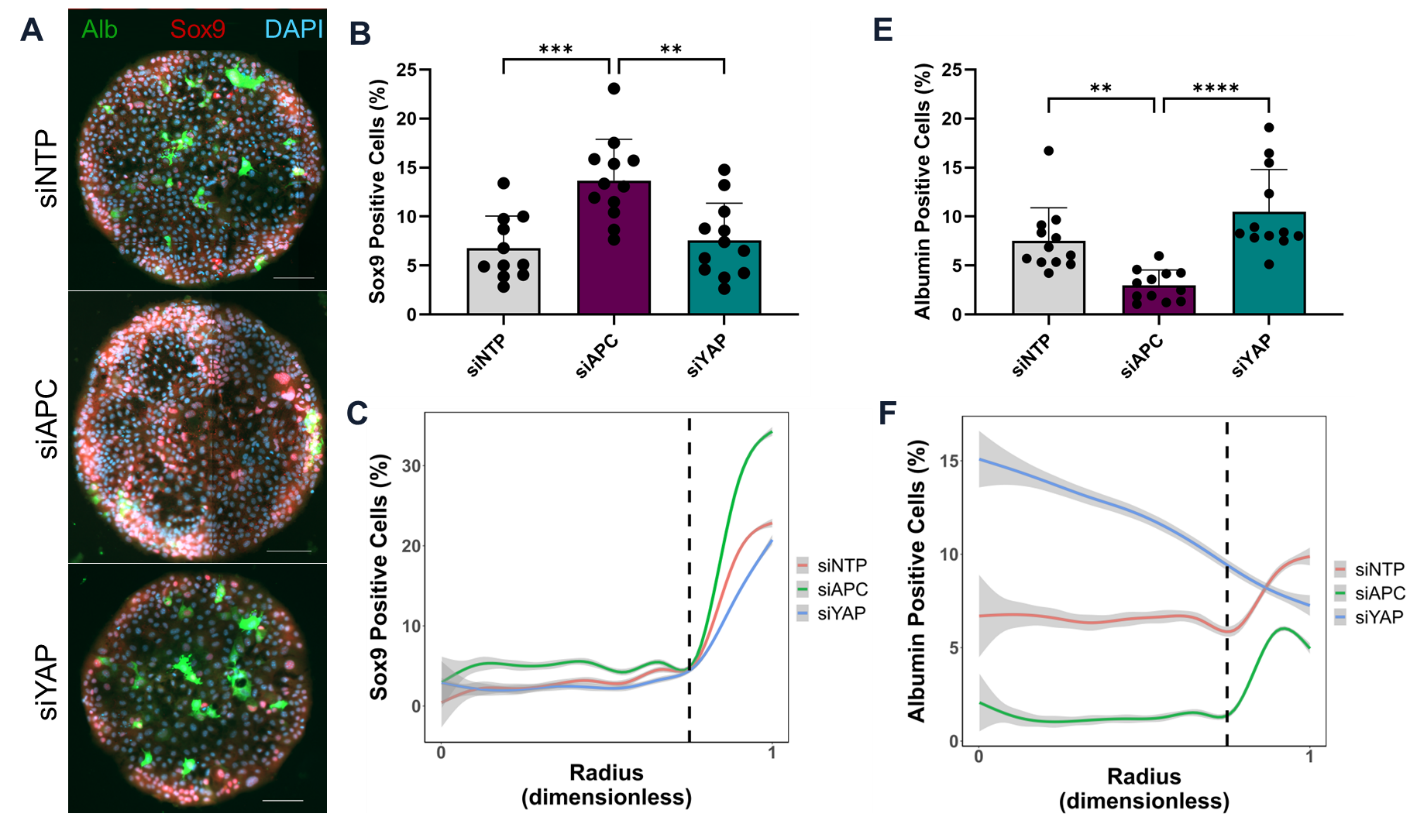

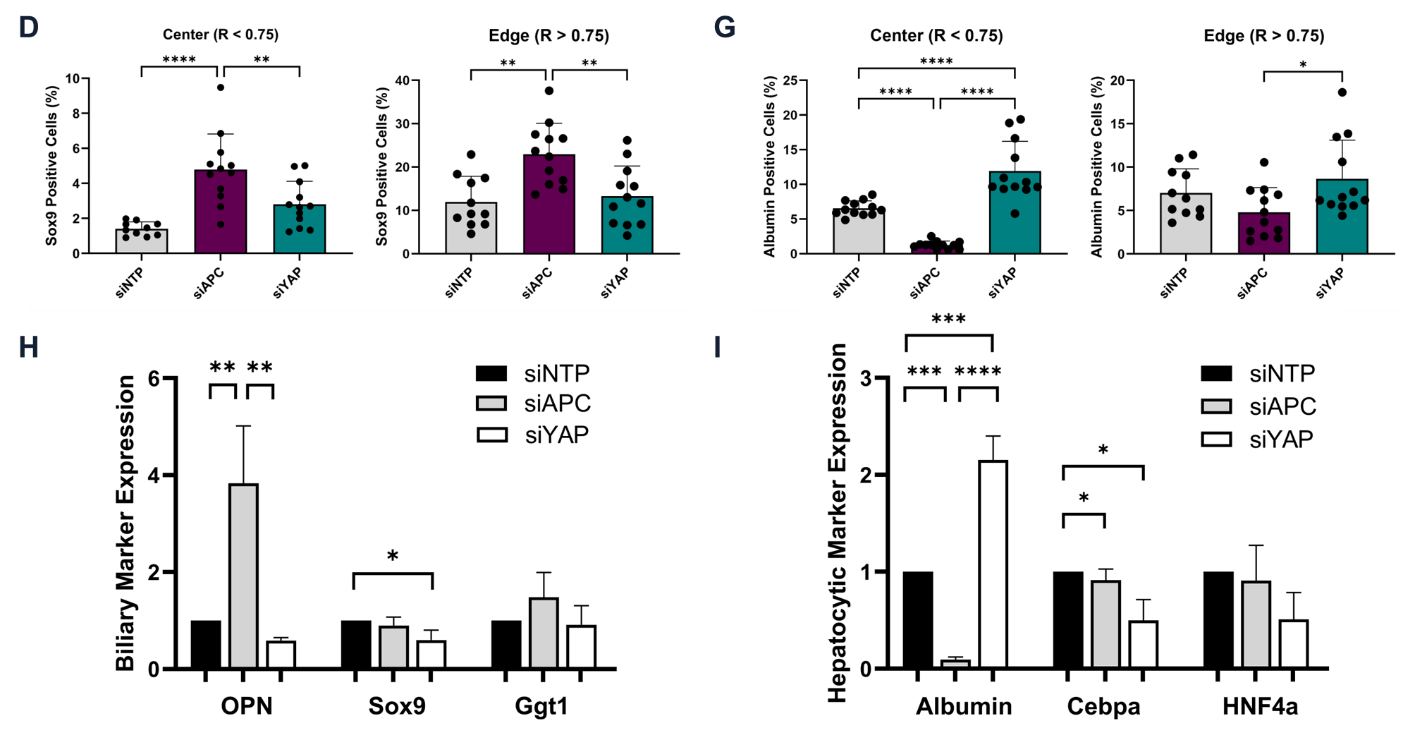


**Supplemental Figure S3** Hepatocytic and biliary marker expression (A) Representative images of differentiated islands treated with siRNA for 72 hours stained for DAPI (blue), Albumin (green), and Sox9 (red). (B&E) Percent Albumin+ and Sox9+ cells. (C&F) Spatial patterning graphs. (D&E) Percent Albumin+ and Sox9+ cells by region. (H&I) Relative gene expression of hepatocytic and biliary markers for siRNA treated cells compared to the control. Statistics are calculated using one-way ANOVA with Tukey’s post-hoc test for bar plots and error bars represent standard deviation. P-values indicated for P < 0.05 (*), P < 0.01 (**), P < 0.001 (***), and P < 0.0001 (****). Scale bars are 100 μm.


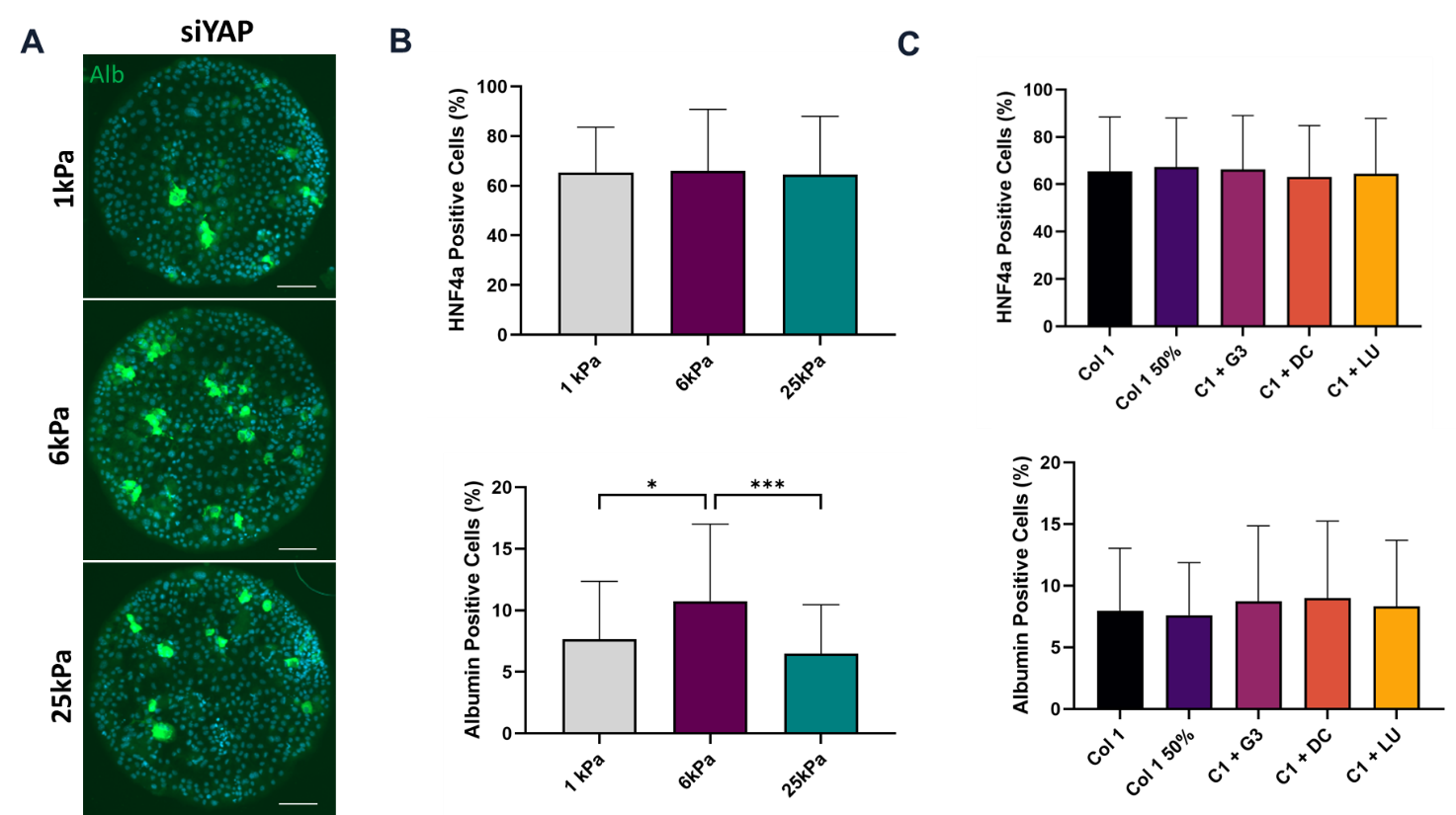


**Supplemental Figure S4** ECM and substrate stiffness modulate hepatocytic expression (A) Representative images of differentiated islands treated with siYAP stained for DAPI (blue) and Albumin (green). (B) Effect of substrate stiffness on percent Albumin+ and HNF4a+ cells for all conditions. (C) Effect of ECM composition on percent Albumin+ and HNF4a+ cells for all conditions. Statistics are calculated using one-way ANOVA with Tukey’s post-hoc test for bar plots and error bars represent standard deviation. P-values indicated for P < 0.05 (*), P < 0.01 (**), and P < 0.001 (***). Scale bars are 100 μm.


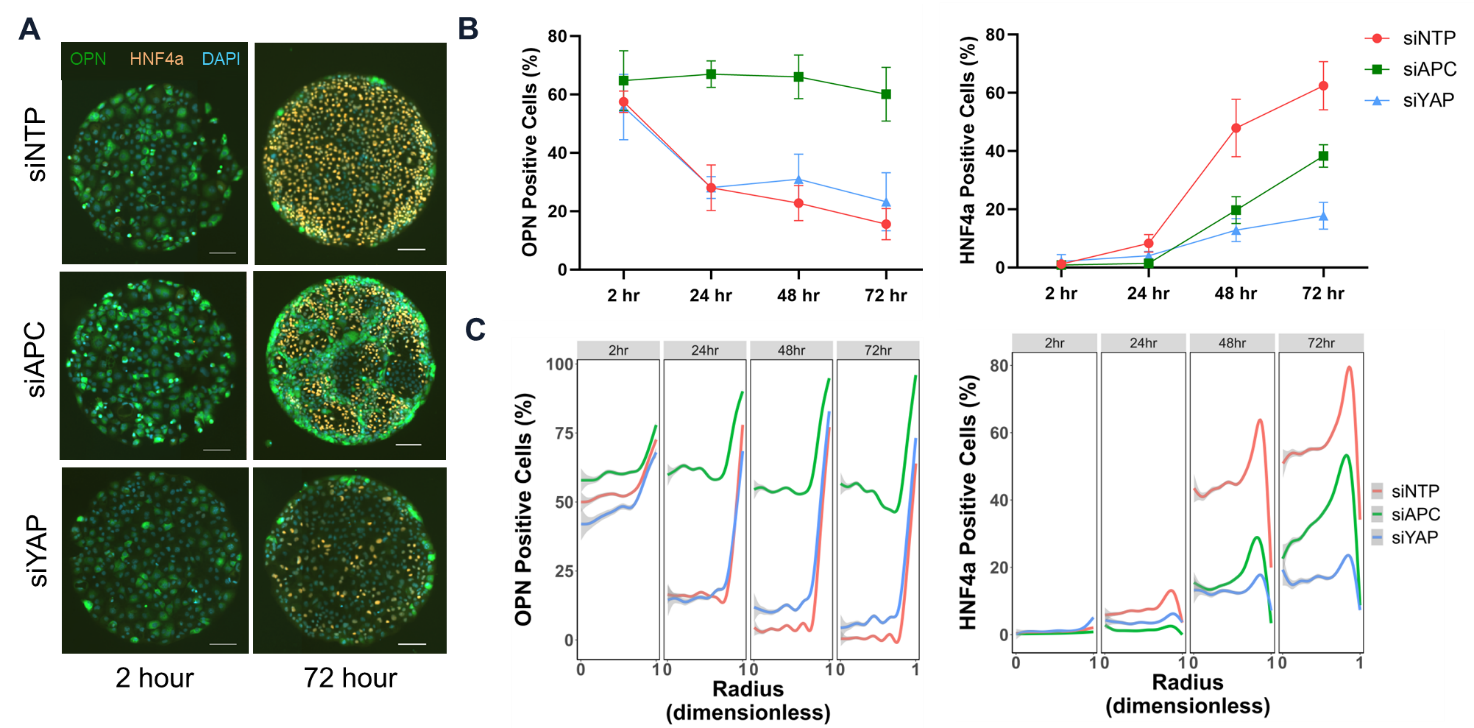


**Supplemental Figure S5** Differentiation Kinetics of APC and YAP Deficient Cells. (A) Representative images of undifferentiated islands treated with siRNA targeting APC and YAP stained for DAPI (blue), HNF4α (red), and OPN (green) at the 2 and 72 hour timepoints. (B) Mean Percent OPN+ and HNF4α+ cells for siRNA knockdowns at 2, 24, 48, and 72 hours. Error bars represent standard error of the mean. (C) Spatial patterning graph of OPN+ and HNF4α+ cells across timepoints. Scale bars are 100 μm.

**
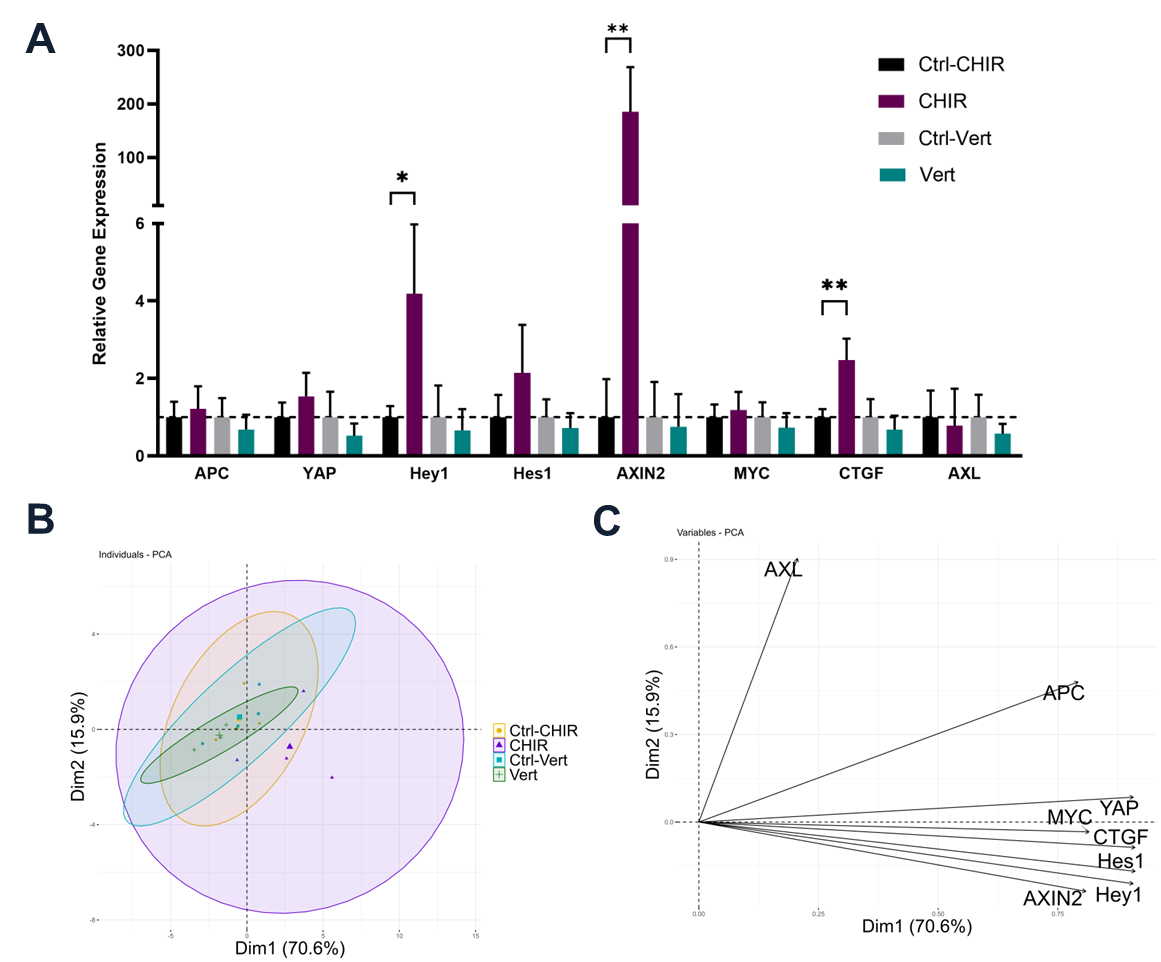
**

**Supplemental Figure S6** Gene Expression Analysis (A) Relative gene expression of CHIR99021 and verteporfin treated cells normalized to DMSO treated control cells. (B&C) Biplot and loading vectors for the relative gene expression plotted on PC1 and PC2. Statistics are calculated using unpaired t-test for bar plots and error bars represent standard deviation. P-values indicated for P < 0.05 (*), P < 0.01 (**), and P < 0.001 (***).


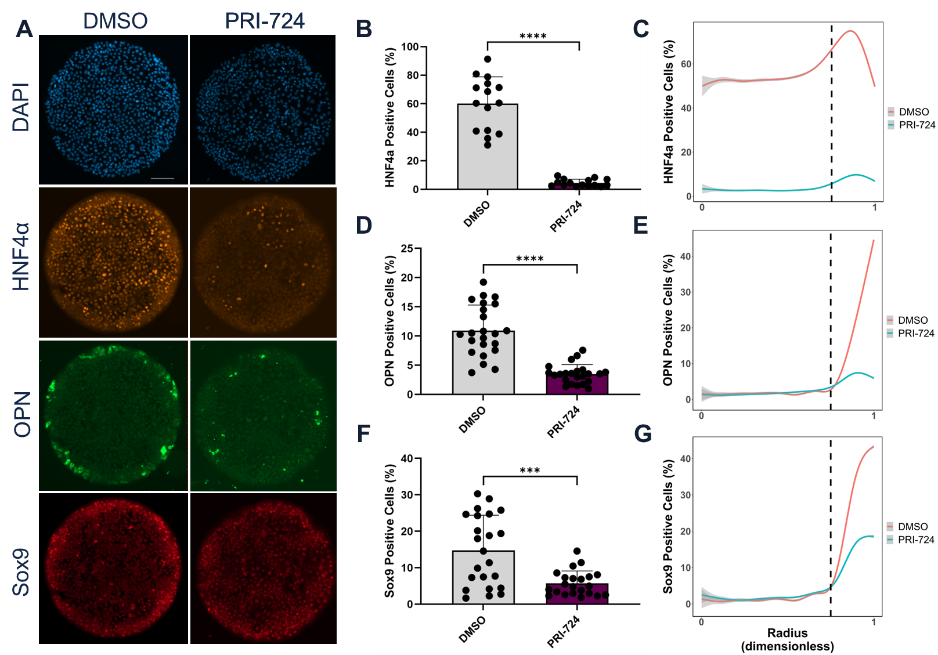

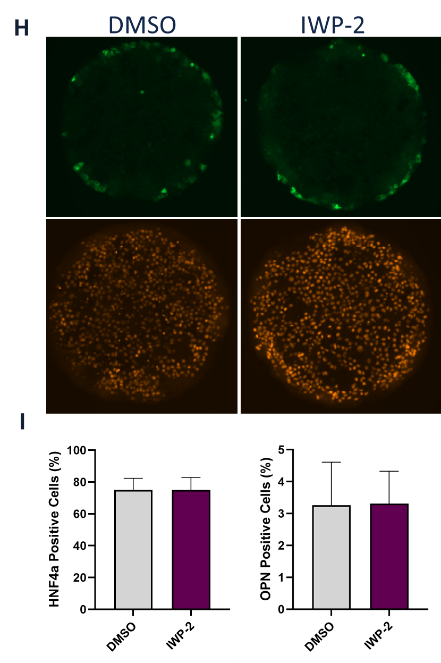


**Supplemental Figure S7** Wnt Inhibition (A) Representative images of differentiated islands treated with PRI-724 for 72 hours stained for DAPI (blue), OPN (green), HNF4a (orange), and Sox9 (red). (B-G) Bar and line graphs showing percent positive cells and spatial patterning for cells treated with PRI-724. (H) Representative images of islands treated with IWP-2 and stained for HNF4a (orange) and OPN (green). (I) Bar graphs showing percentage of OPN and HNF4a positive cells when treated with IWP-2. Statistics are calculated using unpaired t-test for bar plots and error bars represent standard deviation. P-values indicated for P < 0.05 (*), P < 0.01 (**), P < 0.001 (***), and P < 0.0001 (****). Scale bars are 100 μm.


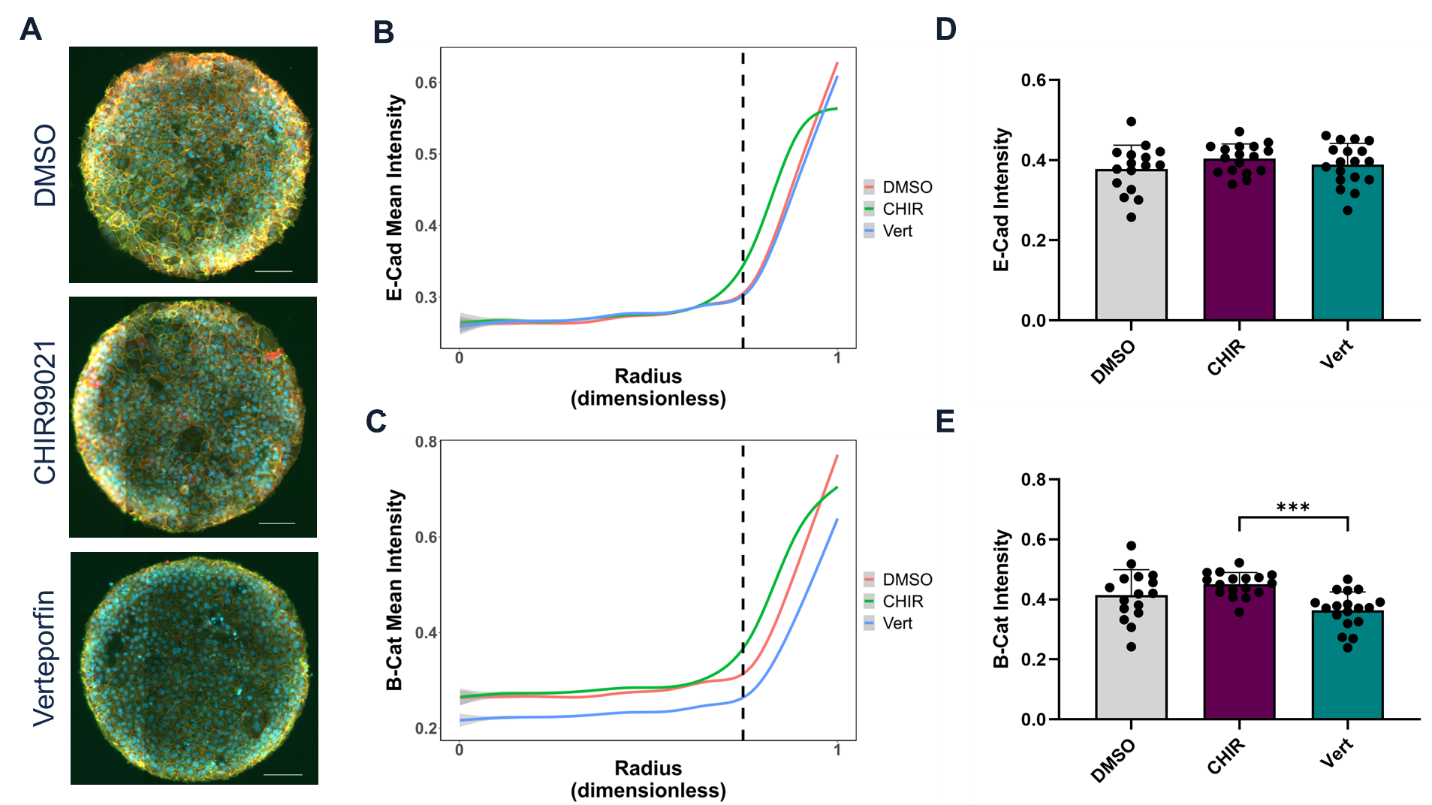


**Supplemental Figure S8** Adherence Junction Protein Expression (A) Representative images of islands stained for DAPI (blue), β-Catenin (red), and E-Cadherin (green). (B&D) Radial plots showing E-Cadherin & β-Catenin expression levels. (C&E) Bar graphs for E-Cad & β-Cat expression. Statistics are calculated using one-way ANOVA with Tukey’s post-hoc test for bar plots and error bars represent standard deviation. P-values indicated for P < 0.05 (*), P < 0.01 (**), and P < 0.001 (***). Scale bars are 100 μm.


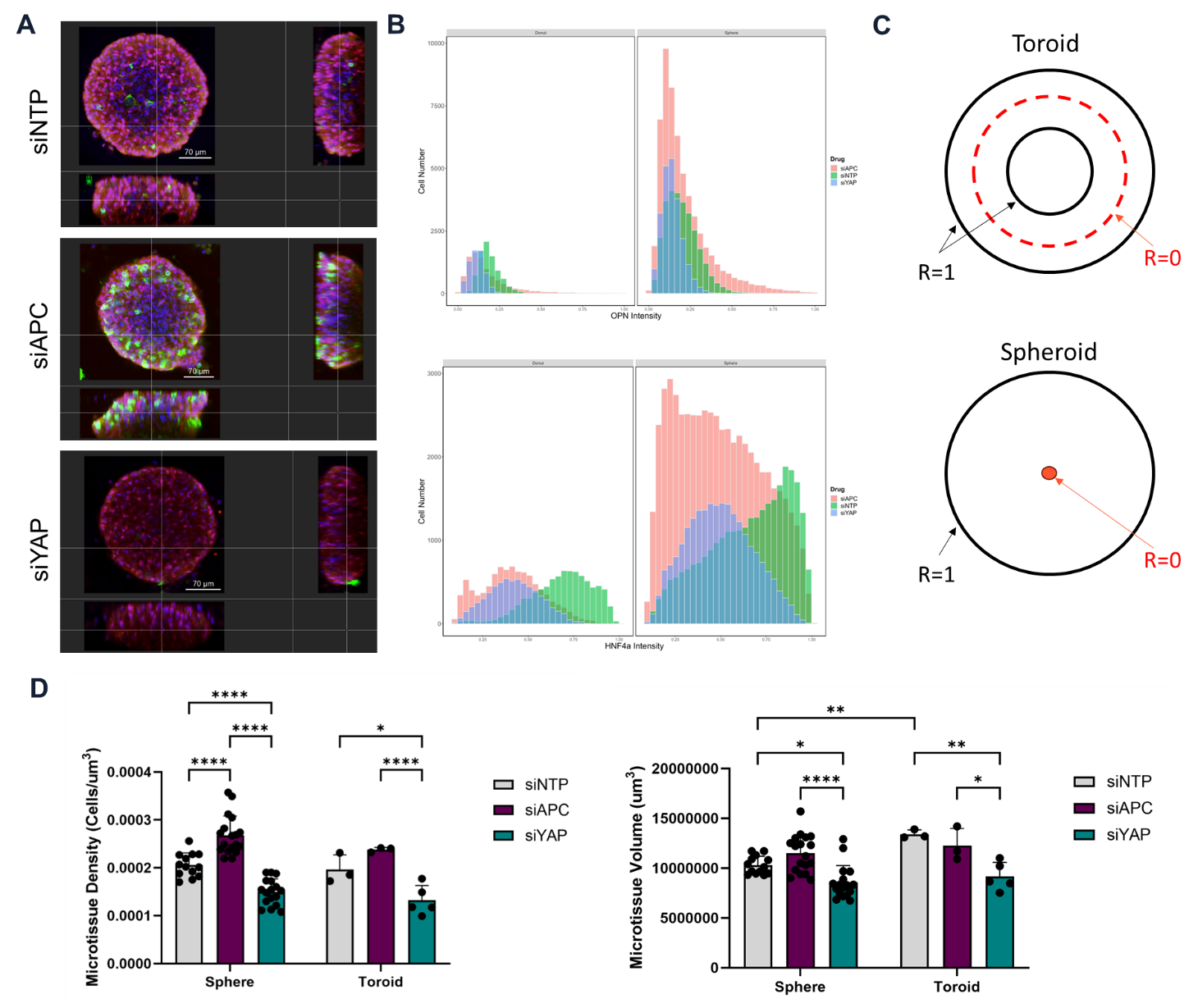


**Supplemental Figure S9** (A) Cross section images of spheroids stained for DAPI (blue), OPN (green), and HNF4α (red). (B) Histogram of immunofluorescent intensities for the microtissues. (C) Diagram of an XY cross-section for a toroid and spheroid showing the center of the microtissue is R=0 while the surface is R=1. (D) Volume and cellular density of the microtissues.

Supplemental Table S1: RT-qPCR Primer Sequences

*
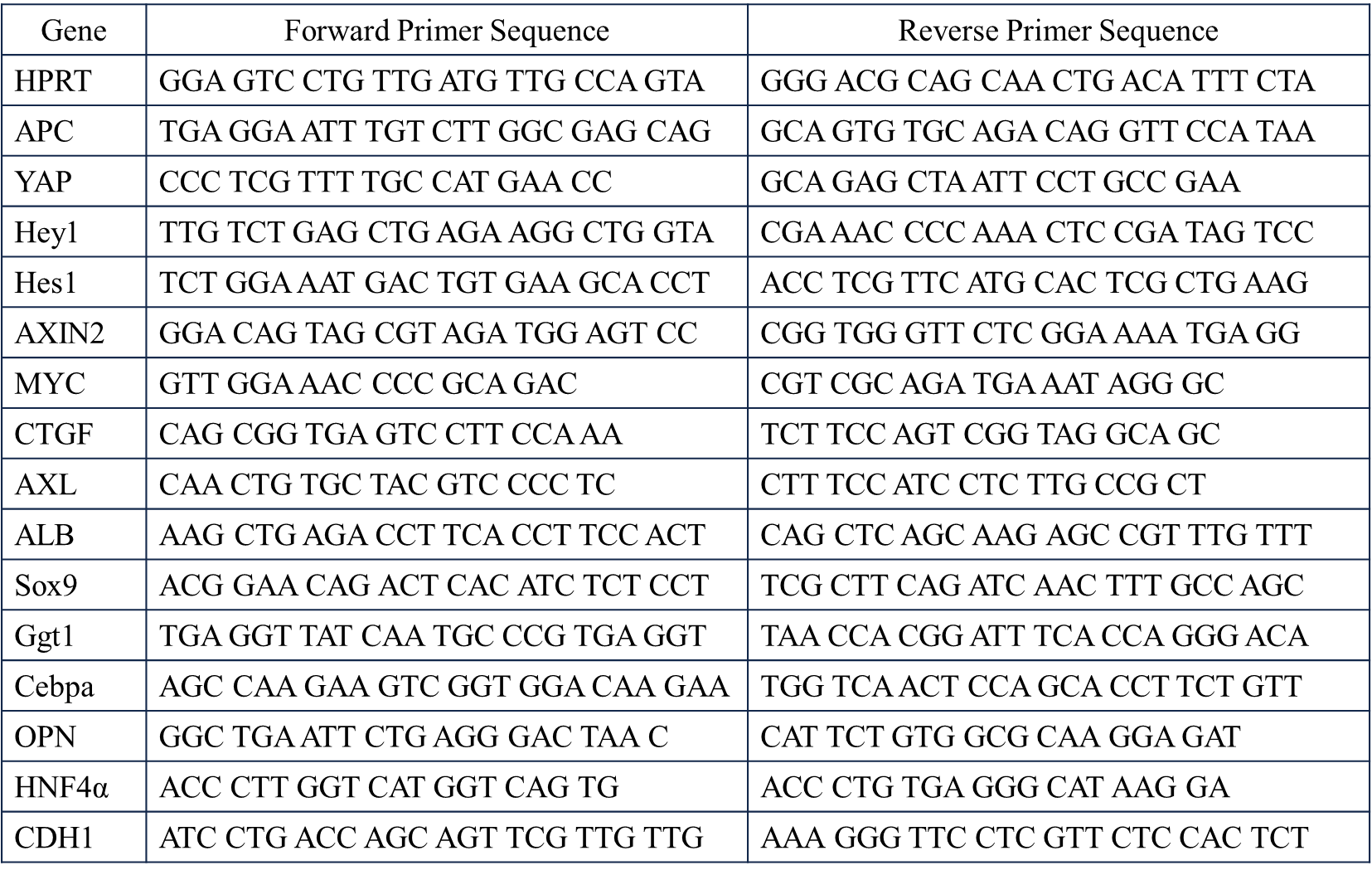
*

Supplemental Table S2: siRNA Sequences


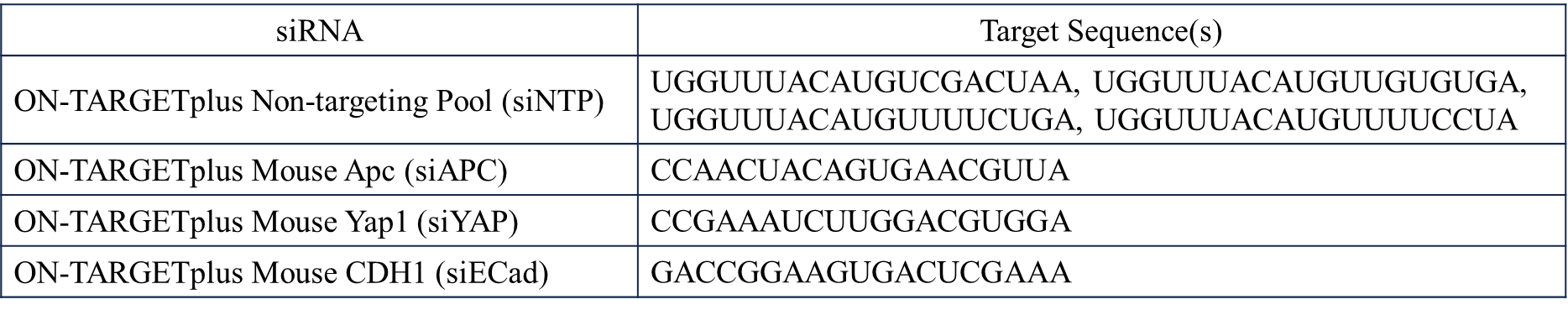

Supplement: Multimedia component 1 [file mmc1.docx]
